# Target Genes of c-MYC and MYCN with Prognostic Power in Neuroblastoma Exhibit Different Expressions during Sympathoadrenal Development

**DOI:** 10.3390/cancers15184599

**Published:** 2023-09-16

**Authors:** Ye Yuan, Mohammad Alzrigat, Aida Rodriguez-Garcia, Xueyao Wang, Tomas Sjöberg Bexelius, John Inge Johnsen, Marie Arsenian-Henriksson, Judit Liaño-Pons, Oscar C. Bedoya-Reina

**Affiliations:** 1 Department of Microbiology, Tumor and Cell Biology (MTC), Biomedicum, Karolinska Institutet, SE-171 65 Stockholm, Sweden; 2 Paediatric Oncology Unit, Astrid Lindgren’s Children Hospital, SE-171 64 Solna, Sweden; 3 Department of Women’s and Children’s Health, Karolinska Institutet, SE-171 77 Stockholm, Sweden

**Keywords:** neuroblastoma, c-MYC, MYCN, prognosis, gene signature, sympathoadrenal development

## Abstract

**Simple Summary:**

More than 40% of high-risk neuroblastoma (NB) cases are characterized by aberrations in the transcription factors MYCN and c-MYC. Nevertheless, the developmental stage and mechanisms through which MYCN and c-MYC contribute to the onset and progression of the malignancy are not fully understood. In this study, we selected different c-MYC and MYCN targets with clinical or biological relevance and prognostic value to model multigene transcriptional risk scores. We found that the modeled scores accurately classify patients into groups with different outcomes, with high-risk-scoring patients exhibiting worse clinical features. Furthermore, target genes associated with poor prognosis showed a higher expression in sympathoblasts than in chromaffin cells during the sympathoadrenal development.

**Abstract:**

Deregulation of the MYC family of transcription factors c-MYC (encoded by *MYC*), MYCN, and MYCL is prevalent in most human cancers, with an impact on tumor initiation and progression, as well as response to therapy. In neuroblastoma (NB), amplification of the *MYCN* oncogene and over-expression of *MYC* characterize approximately 40% and 10% of all high-risk NB cases, respectively. However, the mechanism and stage of neural crest development in which MYCN and c-MYC contribute to the onset and/or progression of NB are not yet fully understood. Here, we hypothesized that subtle differences in the expression of MYCN and/or c-MYC targets could more accurately stratify NB patients in different risk groups rather than using the expression of either *MYC* gene alone. We employed an integrative approach using the transcriptome of 498 NB patients from the SEQC cohort and previously defined c-MYC and MYCN target genes to model a multigene transcriptional risk score. Our findings demonstrate that defined sets of c-MYC and MYCN targets with significant prognostic value, effectively stratify NB patients into different groups with varying overall survival probabilities. In particular, patients exhibiting a high-risk signature score present unfavorable clinical parameters, including increased clinical risk, higher INSS stage, *MYCN* amplification, and disease progression. Notably, target genes with prognostic value differ between c-MYC and MYCN, exhibiting distinct expression patterns in the developing sympathoadrenal system. Genes associated with poor outcomes are mainly found in sympathoblasts rather than in chromaffin cells during the sympathoadrenal development.

## 1. Introduction

With around 13 cases per million and representing 7% of all pediatric cancers worldwide, neuroblastoma (NB) is the most common malignant extracranial solid tumor during early childhood. Despite affecting a relatively small proportion of children, this malignancy is responsible for 15% of all pediatric cancer deaths [1,2]. Neuroblastoma is a heterogeneous disease with clinical presentations from spontaneous regression and differentiation to high-risk cases with metastatic spread and fatal outcomes despite intensive multimodal therapies [3,4,5,6]. Although tumors in children present a low mutational rate in comparison to adult neoplasms [7], specific chromosomal aberrations (1p and 11q loss and 17q gain) [8,9], mutations in certain genes (*ALK*, *PHOX2B*, *ATRX*, and *TERT*), and amplification of the *MYCN* oncogene are linked to NB tumorigenesis and outcome [10,11]. 

Risk stratification of NB patients by the Children’s Oncology Group (COG) and the INRG International Neuroblastoma Risk Group (INRG) supports tailored therapies in association with outcome prediction. To classify patients in risk groups, COG employs post-surgical clinical information [12,13,14] and INRG uses pre-treatment tumor imaging and clinical criteria, including *MYCN* amplification status [15]. Even with multiple staging systems in place, there is room for development, especially among high-risk patients whose long-term survival rate is only around 50% [16,17,18]. Furthermore, a substantial proportion of high-risk patients experience a range of side effects due to the high toxicity of conventional chemotherapy. Thus, more detailed stratification systems and the identification of valuable biomarkers are needed to make targeted therapies available for NB patients, providing more effective and less harmful treatment options [17,18].

*MYCN* amplification is among the best predictors of poor outcomes in NB [19,20]. The aberrant activity of the MYC family of transcription factors, which include c-MYC, MYCN, and MYCL, is frequently observed in cancer [19,21,22]. Both c-MYC and MYCN play pivotal roles in regulating key biological processes, including cell proliferation, apoptosis, differentiation, and senescence [19,23,24,25,26,27,28]. In development, the expression of *MYCN* and *MYC* is delicately regulated. While *MYCN* is preferentially expressed in neural tissue and during early stages [29], *MYC* has a more ubiquitous expression across various tissues and cell types [30]. A distortion in the expression of *MYCN* and *MYC* can have severe phenotype consequences. An elevated expression of *MYC* in the primitive streak or *MYCN* in undifferentiated mesoderm significantly affects the normal ontogeny of these germ layers [31,32]. Furthermore, an aberrant expression of *MYCN* in neural crest cells induces NB in mouse and zebrafish models [33,34]. The deregulated activity of both c-MYC and MYCN has also been shown to play a critical role in maintaining a stem-like state in cancer cells by blocking differentiation pathways and promoting the expression of self-renewal and pluripotency genes [35,36,37]. In consequence, the upregulation of MYCN and c-MYC target genes is a characteristic feature of the progression of aggressive NB, with c-MYC as the main driver in INSS stage 4 non-*MYCN*-amplified tumors [38].

Approximately 20% of all NB cases, and 40% of high-risk patients, present *MYCN* amplification [22]. While *MYCN* amplification results in its over-expression [39], an aberrant activity of MYCN has also been implicated in the development of high-risk NBs in a manner independent of *MYCN* copy number [38,40]. For example, the upregulation of the FOXR2 protein was shown to enhance MYCN stability, leading to a more aggressive form of the disease [40]. Additionally, the RNA-binding protein LIN28B has been shown to augment *MYCN* mRNA abundance by suppressing let-7 microRNA biogenesis, resulting in increased MYCN levels, even in the absence of *MYCN* amplification [41,42]. Similarly, Zimmerman et al. showed enhanced *MYC* expression in a subset of NB due to focal enhancer amplification or enhancer hijacking. Based on these findings, the authors defined a subset of high-risk NB patients (~10%) that clinically resembled cases with *MYCN* amplification [43]. Furthermore, an *MYCN* gene-signature identified from 88 NB patients provided powerful outcome prediction in the absence of *MYCN* amplification [44]. Given these findings, we asked whether the additive effect of subtle differences in the expression of MYCN and c-MYC target genes could contribute to further defining high-risk NB cases, even in the absence of *MYCN* amplification or over-expression of the genes.

Here, we developed an analytical framework aimed at determining c-MYC/MYCN target genes with prognostic value in NB using LASSO-penalized Cox (i.e., LASSO-Cox) models [45]. To this end, we studied the prognostic power of c-MYC/MYCN target genes obtained by various screening approaches. Additionally, we investigated the expression patterns of these target genes in the developing sympathoadrenal system. Our data provides insights into their potential implications for risk classification and disease progression in NB, and ultimately about its cell of origin for early diagnosis and treatment.

## 2. Materials and Methods

### 2.1. c-MYC/MYCN Target-Gene Sets

Three c-MYC/MYCN target sets were included for the analysis. The first was derived from the hallmark gene set available in the Molecular Signatures Database of GSEA (https://www.gsea-msigdb.org/, accessed on 13 April 2023), which comprises curated gene datasets obtained from previously published studies [46]. In particular, the ‘HALLMARK_MYC_TARGETS_V1′ and ‘HALLMARK_MYC_TARGETS_V2′ gene sets contain 200 and 58 MYC-regulated genes, respectively [47]. After removing duplicated genes and merging the two sets, a set of 240 genes was obtained (i.e., c-MYC). The second set of target genes (i.e., c-MYC ChIP) was generated in a previous study through a combination of chromatin immunoprecipitation (ChIP) and promoter microarrays, identifying a total of 1469 c-MYC direct target genes detected in HeLa cells and human fibroblast [48]; 1459 of these genes were not redundant and had an expression reported in the SEQC cohort. It should be noted that the c-MYC and c-MYC ChIP targets originated from diverse resources and methodologies, encompassing different summaries and experimental viewpoints. The third target set (i.e., MYCN) consisted of 157 genes generated by both identifying downstream targets of MYCN through shRNA-mediated silencing of *MYCN* in NB cells and by selecting genes with an expression profile correlated with *MYCN* mRNA levels in a series of 88 NB samples. The validation of the MYCN target-gene set was also performed using a ChIP-on-chip assay [44]. The original gene set lists are provided in Supplementary Table S1.

### 2.2. Expression Analysis of NB Cohorts

**Figure 1:**
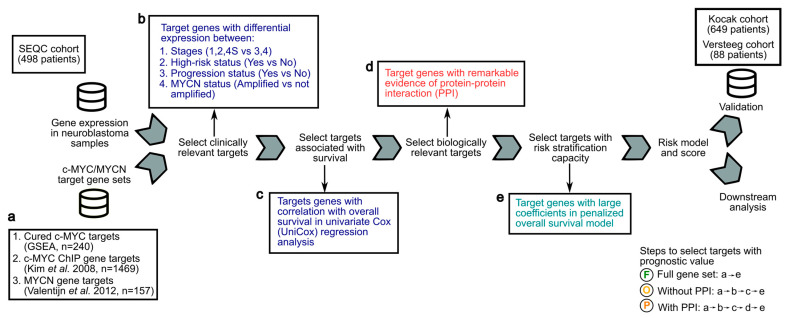
Analytical framework to screen c-MYC/MYCN target genes with prognostic value for the development of a risk-score model for NB patients. Gene expression and clinical data from the SEQC cohort of 498 NB patients were analyzed to compute a transcriptional risk score that summarizes the effect of multiple c-MYC/MYCN target genes with prognostic value. (**a**) c-MYC targets were obtained from a curated database (GSEA) [47] and from a previous investigation conducted using ChIP [48]. MYCN targets were obtained from a study conducted using experimental and computational methods [44]. These targets (i.e., “Full gene set”) were further filtered to select genes correlated with survival, presenting biological and/or clinical relevance, regardless of, or exhibiting remarkable interactions (i.e., “Without protein-protein interaction PPI”, respectively). The resulting datasets and the steps to obtain them are illustrated with an encircled “F” (i.e., Full gene set, produced by steps “a-e”), “O” (i.e., without PPI, produced by steps “a-b-c-e”), and “P” (i.e., with PPI, produced by steps “a-b-c-d-e”). (**b**) Targets with clinical relevance were selected using as a proxy the significance of their expression difference between patients (1) in high- and not-high-risk groups, (2) in high and low INSS stages, (3) with different progression status, and (4) with different *MYCN* amplification status (Wilcoxon rank test, two-tail, FDR < 0.05). (**c**) Targets with an expression correlated with overall survival were determined by a univariate Cox model and further selected. (**d**) Targets interacting with others were selected using remarkable evidence from the literature (STRING confidence score > 0.9) [58]. (**e**) Selected targets with prognostic value were determined using LASSO-Cox regression, and a risk score for each patient was then computed using the expression of these targets and the coefficients from the model. The prognostic prediction capabilities of this risk score to stratify patients into two risk groups were further validated in the Kocak [52] and Versteeg cohorts [53] by survival analysis and dimension reduction via t-distributed stochastic neighbor embedding (t-SNE).

To study the expression of the c-MYC/MYCN gene sets in NB, RNA sequencing data matrices and their corresponding clinical information (i.e., gender, age, INSS stage, risk status, *MYCN* status, progression status, favorable outcome status, survival time, and survival status) were obtained from the R2 database (https://r2.amc.nl/, accessed on 18 April 2023). Specifically, the raw counts (reads per millions, RPMs) for genes and patients in the SEQC cohort (n = 498, GEO accession GSE47774, [49,50]) were obtained and transformed using log2(RPMs + 1). These values were used to develop a risk-score signature model and conduct other downstream analysis, as illustrated in Figure 1. The SEQC cohort employs risk classification to group patients based on the following specific criteria. High-risk cases have either been diagnosed with stage 4 disease for over 18 months or have tumors with *MYCN* amplification, regardless of age or stage [51]. For validation, we employed the Kocak (n = 649, GEO accession GSE45547, [52]) and Versteeg (n = 88, GEO accession GSE16476, [53]) cohorts. Briefly, gene expression computed for the Versteeg cohort was obtained with the Affymetrix Human Genome U133 Plus 2.0 microarray and normalized using MAS5.0 in the GCOS software with trimmed mean 96 set to 100 (alpha1 = 0.04, alpha2 = 0.06, [53]). The expression for the Kocak dataset was obtained using single-color gene expression profiles from 649 neuroblastoma tumors using 44K customized oligonucleotide microarrays and normalized using the quantile algorithm from limma [52,54]. The normalized gene expression matrices and available clinical information for both cohorts were obtained from the R2 platform (https://r2.amc.nl/, accessed on 18 April 2023). Gene expression was further log2(x + 1) transformed for the various analyses (where “x” refers to the normalized values). Note that the Kocak cohort does not include a clinical risk assessment of patients. The survival probabilities for this cohort were kindly provided by Professor Matthias Fischer (DKFZ).

### 2.3. Analysis of the Prognostic Value of c-MYC/MYCN Target Genes in NB

To investigate the potential of c-MYC/MYCN-target-gene expression in risk stratification and survival prediction for NB, we employed four different gene screening approaches in the SEQC cohort (n = 498) [49,50,51]. Further, the predictions were validated using the Kocak (n = 649) [52] and the Versteeg cohorts (n = 88) [53]. We conducted these screenings with the purpose of focusing our analysis on genes with clinical and/or biological relevance, and to facilitate a more accurate selection of genes with prognostic value. In detail, when the number of genes measured largely exceeds the number of patients, the Cox proportional hazard models linking expression to patient survival results in a lack of stability and overfitting [55,56]. Gene pre-filtering in combination with LASSO regression have shown promising results in mitigating this problem [55,56].

The first approach is aimed at identifying differential expression patterns of c-MYC/MYCN targets between NB patients with poor and better outcomes, and to select targets with clinical relevance. Specifically, we identified differentially expressed genes (DEGs) between patients in (1) high (i.e., 3 and 4) and low (i.e., 1, 2, and 4S) INSS stage, and between patients with (2) different risks, (3) progression, and (4) *MYCN* status, using two-tailed Mann-Whitney U/Wilcoxon rank-sum tests. To ensure the reliability and validity of our results, we used the Benjamini-Hochberg (FDR) method [57] to adjust the *p*-values and control the false discovery rate (FDR) at a threshold of 0.05. In the second approach, the correlation between the expression of c-MYC/MYCN target genes and overall patient survival using univariate Cox regressions was examined. Genes with an FDR of less than 0.05 were considered significantly correlated with overall survival. 

For the third approach, genes encoding proteins with remarkable evidence of interaction were identified by protein-protein interaction (PPI) analysis using the STRING database (https://string-db.org/, version 12.0, accessed on 18 April 2023). STRING aggregates interaction data from various sources, including text mining, experimental studies, and computational methods based on co-expression and shared genomic context [58]. A PPI network for the genes in the SEQC cohort was constructed with their proteins as nodes and STRING PPI confidence scores as weighted edges. In this network, PPIs with a confidence level lower than 0.9, indicating a 90% likelihood that two proteins appear in a single KEGG pathway, were excluded. Similarly, proteins (i.e., nodes) with a degree value of zero, indicating they have no remarkable interactions with other proteins in the network, were filtered out. Genes independent of the remarkable interaction status in the PPI network were labeled as “Without PPI”, while those with significance were labeled as “With PPI”. Note that the genes resulting from the “With PPI” screening are a subset of those in the “Without PPI” gene set. The objective of this approach was to select and analyze significant protein interactions within the network as a proxy of target genes with a strong biological connection (i.e., for instance, those involved in the same molecular pathways).

The fourth approach was based on using the least absolute shrinkage and selection operator (LASSO) performing Cox regression model to screen for c-MYC/MYCN target genes with prognostic value and identifying optimal weighting coefficient scores (i.e., LASSO β coefficient). The LASSO penalty was used to eliminate genes with limiting contribution and select the most influential ones, with the optimal tuning parameter λ selected through cross-validation. The tuning parameter λ controls the strength of the penalty, with larger values resulting in more genes being eliminated. c-MYC/MYCN target genes with prognostic power, and their corresponding LASSO β coefficient, were established using the optimal log λ score with the ‘glmnet’ package [45,59]. The risk score for each sample was calculated with the computed LASSO β coefficient and the expression value for each gene using the equation risk score = $\sum_{i=1}^{n}\left({Expression}_{i}\times \beta_{i}\right),$ where *i* represents a gene in the set of *n* influential genes with prognostic power. The median value of the risk scores for the patient cohort was used as a cutoff for the prognostic tool to evaluate overall survival.

Note that for all the screening approaches we used statistical tests in the normalized and transformed gene expression matrix. We favored this approach over using the raw data in combination with more sophisticated algorithms to determine DEGs [60,61]. In this way, the same data could be used in every screening step. Furthermore, in some instances, the Mann-Whitney U/Wilcoxon rank-sum test has shown better control for false discoveries compared to more sophisticated approaches [62].

### 2.4. Prognostic Power Evaluation by Survival and Efficiency Analysis 

The 498 samples in the SEQC cohort were divided into two risk groups based on their computed c-MYC/MYCN risk score. Patients with a high-risk score (HR, *n* = 249) presented a risk score higher than the median, while patients with a low-risk score (LR, *n* = 249) presented a risk score lower than the median. To thoroughly evaluate the prognostic power of the computed risk score, four distinct methods were used: (i) Log-rank tests of survival were conducted between the low- and high-risk-score groups. This analysis allowed the comparison of survival curves between the two groups to determine the significance of the survival differences. (ii) The area under the curve (AUC) of the receiver operating characteristic (ROC) was used to assess the efficiency and accuracy of the prognostic model. Both of these values were computed using the ‘survivalROC’ package, which generates a time-dependent ROC curve from censored survival data with the Kaplan-Meier method [63]. A higher AUC value indicates a better performance of the prognostic model in predicting patient survival. (iii) t-distributed stochastic neighbor embedding (t-SNE) was utilized to visualize the distribution of the different clinical risk groups defined by SEQC as well as c-MYC/MYCN risk groups based on the expression of the selected genes generated by the model. (iv) The significance of the differences in risk scores among various clinical outcome groups such as clinical high risk (yes versus no), INSS stage, progression (yes versus no), *MYCN* amplification (yes versus no), favorable outcome (yes versus no), age (older or younger than 18 months), and gender (female versus male) was computed using two-tailed Mann-Whitney U or Kruskal-Wallis tests. 

In determining genes with prognostic value, some clinical variables can have a large effect in the risk stratification and in our results. These variables include treatment regimens and response to therapy. We studied the effect of two groups of clinical covariates available for the SEQC cohort: (1) “d_fav_all” as a proxy of response to therapy, and (2) “d_fav_all”, age at diagnosis and gender. “D_fav_all” reports either “deceased despite chemotherapy” or “event-free without chemotherapy for at least 1000 days post diagnosis” [50]. This variable is available for 272 out of 498 patients. For these groups of variables, the overall survival of patients was modeled using multivariate Cox regression as the result of the expression in interaction with the clinical covariates (i.e., survival~expression + clinical variables). Genes contributing with a significantly different survival in patients (accounting for clinical covariates) were further investigated.

### 2.5. Cross-Species Comparisons and Gene Ontology Analysis 

Gene expression comparisons between human and mice during sympathoblast development and in disease were conducted by selecting only orthologue genes. Specifically, the 1:1 human:mouse high confidence orthologues annotated by ENSEMBL version GRCh38.p12 also present in the comprehensive GENCODE annotations, 28 for humans and 18 for mice, were obtained from the UCSC browser on 25.02.2019. To investigate the molecular function profiles on c-MYC/MYCN targets with prognostic power, gene enrichment analyses for each target set were performed utilizing gene ontologies (GO) annotated by ENSEMBL version GRCh38.p12. Benjamini-Hochberg FDR corrected one-tail Fisher exact tests were used. From the resulting target-gene sets, the names of five genes (out of 117) were manually curated to make them compatible with the ENSEMBL annotation (i.e., *C1orf114* to *CCDC181*, *EIF2C4* to *AGO4*, *KDELC1* to *POGLUT2*, *MKL1* to *MRTFA*, and *WBSCR22* to *BUD23*). Additionally, ten genes (out of 117) lacked high confidence 1:1 orthologues between mouse and human and were not considered for the murine sympathoadrenal development comparisons (i.e., *APBB3*, *G6PD*, *HIST1H4B*, *NOP16*, *NPM1*, *RAD50*, *RAVER1*, *SPCS3*, *UBL5*, and *UBE2L3*).

### 2.6. Single Cell Analysis of Gene Expression Profiles during Sympathoadrenal Development and NB

Single-cell datasets from the sympathoadrenal system were obtained from Furlan et al. (2017) [64] and Jansky et al. (2021) [65], while datasets from NB patients were collected from van Groningen et al. (2017) [66], Kildisiute et al. (2021) [67], and Bedoya-Reina et al. (2021) [68]. PAGODA-processed data from Furlan et al. (2017) [64], including clustering and normalized gene expression magnitude for the developing murine adrenal anlagen at E12.5 and E13.5, was kindly provided by the authors. Significant gene up-regulation was computed for each cluster in comparison to all the others using Benjamini-Hochberg corrected FDRs on one-tailed Welch’s *t*-tests. Genes with an FDR < 0.01 were selected for further analysis. For the human developing adrenal anlagen [65], genes significantly up-regulated in each cluster were kindly provided by Jansky et al. (2021) and are presented in Supplementary Table S5 of the original publication [65]. For the NB datasets, markers of each tumor cell cluster identified for the GOSH cohort and sequenced with 10x by Kildisiute et al. were obtained from the Supplementary Table S6 [67]. For Bedoya-Reina et al., genes with significant up-regulation in each NB cluster were obtained from Supplementary Data S6 [68]. In all cases, genes with an adjusted *p*-value/FDR < 0.01 (i.e., makers) were regarded as significant and selected for further analysis. Gene names were standardized to the GENCODE annotations, 28 for humans and 18 for mice, and cross-species comparison was conducted as detailed above. The comparison included in total 16,694 1:1 high-confidence orthologues out of 56,754 genes annotated by ENSEMBL, also present in the GENCODE annotation. 

To compare the stratification power of each of the gene sets with those of the c-MYC/MYCN targets, each gene set was processed with the pipeline illustrated in Figure 1 using the “Full gene set” approach. The proportion of patients classified in the high-risk group by the c-MYC/MYCN targets as well as by each gene set was computed, and its significance determined using Benjamini-Hochberg corrected one-tail Fisher exact tests. We also computed a signature score for orthologue c-MYC/MYCN target genes in the developing mouse adrenal anlagen using Scanpy [69]. This signature score calculates the average expression of orthologue c-MYC/MYCN targets per 10,000 per cell +1, minus the average of a (n) randomly selected sets of genes per 10,000 per cell +1 (where *n* = max [# orthologue c-MYC/MYCN targets, 50]). The significance of the signature scores in each cell cluster was computed using Benjamini-Hochberg corrected FDRs on one-tailed Mann-Whitney U tests. 

### 2.7. Statistical Analysis

The Kaplan-Meier survival curves were generated by using the median risk score as a threshold to compare the survival risk difference between the low- and high-risk groups. All statistical analyses were performed as indicated previously using Python (version 3.8) and R (version 4.2.2). The Venn diagram was generated using the interactive Venn diagram viewer “jvenn” [70].

## 3. Results

### 3.1. Development of an Analytical Framework to Screen c-MYC/MYCN Target Genes with Prognostic Value in NB

Using previously defined c-MYC, c-MYC ChIP, and MYCN target genes (Figure 1a), we developed an analytical framework to identify c-MYC/MYCN targets with prognostic values and strong biological functional connections, and evaluated their ability to stratify patients in the SEQC [49,50,51], Kocak [52], and Versteeg [53] cohorts (Figure 1). Our aim was to obtain target sets that exhibited differential expressions across various clinical variables, taking into account both their functional cohesion and the potential prediction enhancement that gene pre-filtering could have in our approach [58]. To achieve this, we selected c-MYC/MYCN target genes with significantly differential expression in key clinical prognostic variables, including risk, INSS stages, progression, and *MYCN* amplification status (Figure 1b). We then identified c-MYC/MYCN target genes with high expression in patients with different overall survival, as determined by univariate Cox regression analysis (i.e., uniCox, (Figure 1c). From this target set, we selected c-MYC/MYCN target genes encoding proteins that exhibited remarkable evidence of interaction, as scored by the STRING database (i.e., protein-protein interaction, PPI, Figure 1d). The screened c-MYC/MYCN target genes were categorized into two distinct groups: one comprising genes irrespective of their interaction (referred to as “Without PPI”) and another consisting of genes with remarkable interactions (referred to as “With PPI”). Importantly, the “With PPI” category is a subset of the “Without PPI” category. This strategy allowed us to identify c-MYC/MYCN target genes that may be involved in common pathways arguably associated with malignancy onset and progression (Figure 1). The computed gene list after each approach is shown in Supplementary Table S2. 

### 3.2. Expression of c-MYC/MYCN Target Genes Stratify NB Patients in Clinical Groups with Different Outcomes

**Figure 2:**
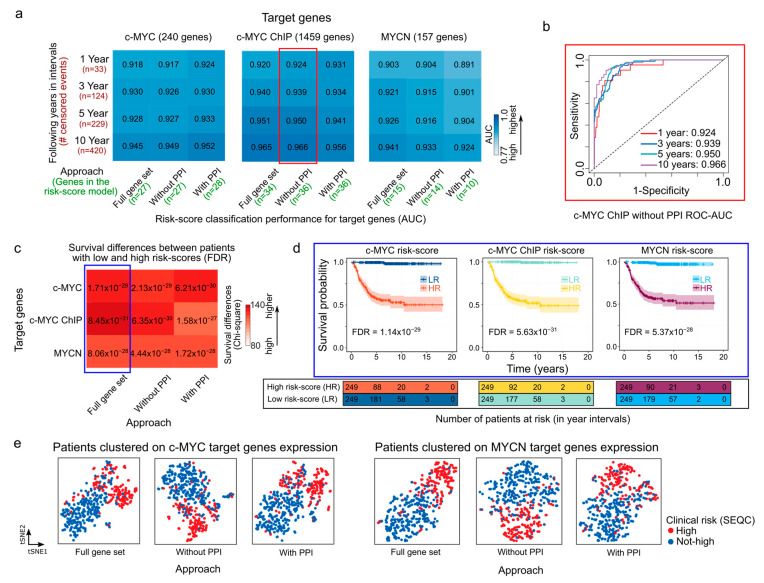
Prognostic performance of risk-score models in NB generated with c-MYC/MYCN target genes. (**a**) Heatmaps showing the performance (AUC values) of the overall survival prediction by risk scores of genes with prognostic value generated by different approaches on c-MYC, c-MYC ChIP, and MYCN target genes. The AUC values were computed using time-dependent ROC curves and indicate the accuracy of the risk scores in distinguishing between patients with varying survival outcomes in different time intervals (i.e., 1, 3, 5, and 10 years). These AUC values are displayed as both values and colors of cells in the heatmap. A higher AUC value represented with bolder blue indicates a better performance of the risk score in predicting patient survival for the SEQC cohort (*n* = 498) [49,50]. The different approaches to select the c-MYC/MCYN target genes (i.e., Full gene set, with and without PPI) are displayed in Materials and Methods and illustrated in Figure 1. (**b**) ROC-AUC plot depicting the predictive performance of the model is displayed for c-MYC ChIP target genes selected using the “Without PPI” approach (surrounded by a red rectangle in (**a**)). (**c**) Survival prediction power of the median risk score computed for genes with prognostic value generated by different approaches on c-MYC/MYCN target genes. The color scale represents the difference between observed and expected deceased patients in two groups: one with high and the other with low median risk scores, as approximated by χ^2^ (Chi-square). Bolder red indicates a larger difference. The expected values are computed assuming an equal number of deceased patients in both groups. FDR-corrected *p*-values [57] indicate the significance of this difference, and the power of the median risk score to predict patients with different outcomes. (**d**) Kaplan-Meier curves exemplify the predictive ability of the model for different targets on the SEQC cohort (surrounded by a blue rectangle in (**c**)). (**e**) t-SNE depicting the transcriptional similarities between patients for genes with prognostic value generated by different approaches on c-MYC and MYCN target genes. Dots representing patients are colored following the SEQC clinical risk classification [51]: high-risk patients are in INSS stage 4 and have at least 18 months at diagnosis or have *MYCN*-amplified tumors. AUC = area under the curve, ROC = receiver operating characteristic, PPI = protein-protein interaction reported by the STRING database [58].

Using as a starting point the c-MYC/MYCN target sets generated by alternating the steps in the previously described analytical framework (as illustrated in Figure 1), we selected c-MYC/MYCN target genes with a strong prognostic power using LASSO-Cox modeling (Figure 1e). This method is widely used [56,71,72,73] and allows for the identification of a reduced number of genes (as illustrated in Figure 2a, at least 10 and at most 36) with a strong association to favorable (β < 0) or poor (β > 0) survival. We identified a specific set of genes with a strong prognostic value for NB, as defined by the β coefficient (Supplementary Table S3). Further, we evaluated the accuracy of the LASSO-Cox models in predicting patient survival using different sets of c-MYC/MYCN target genes (Figure 2a). Our findings suggest that the various approaches used to select c-MYC/MYCN targets prior to the LASSO-Cox modeling step did not significantly impact the prediction accuracy of the selected genes (Figure 2a). In detail, 27, 27 and 28 c-MYC targets were selected by the “Full gene set”, “Without PPI”, and “With PPI” approaches, respectively; 34, 36, and 36 targets were identified from the c-MYC ChIP set with the “Full gene set”, “Without PPI”, and “With PPI” approaches, respectively; and 15, 14, and 10, were determined for MYCN targets with the “Full gene set”, “Without PPI”, and “With PPI” approaches, respectively. For example, c-MYC targets with strong evidence of interaction (i.e., with PPI) exhibited the highest accuracy in predicting risk score in NB patients across all survival time intervals (Figure 2a), whereas MYCN targets with strong evidence of interaction had the lowest accuracy among the different gene sets (Figure 2a). In contrast, c-MYC ChIP targets obtained without PPI filtering ahead of the LASSO-Cox modeling presented the highest accuracy among the different groups (Figure 2b). Notably, the accuracy of the LASSO-Cox models computed using the SEQC cohort (Figure 2a) were verified using the Kocak and Versteeg cohorts (Figures S1 and S2).

We next aimed to calculate a risk score to summarize and evaluate the survival predictions for each patient based on the different c-MYC/MYCN target sets obtained through the different approaches. To this end, we computed a score that considered both target-gene expression and their corresponding β coefficients as provided by the LASSO-Cox model. Further, we assessed the capabilities of this risk score to categorize NB patients into groups with either favorable or unfavorable overall survival outcomes. We observed that patients with high c-MYC/MYCN signature risk scores exhibited poorer survival outcomes compared to those with low-risk scores, regardless of the screening conducted prior to the LASSO-Cox modeling. This distinction is illustrated by χ2 (Chi-square) values indicating the differences between expected and observed survival rates (Figure 2c) and is evident in the survival plots (Figure 2d). The observed survival disparity can be replicated in both the Kocak (Figure S1b) and Versteeg cohorts (Figure S1d). Using t-SNE, we then clustered patients based on the expression of c-MYC/MYCN target genes with prognostic power (i.e., those selected by the LASSO-Cox model). The t-SNE plots revealed that the clustering reflected the clinical risk classification obtained from the SEQC dataset (Figure 2e). In particular, the t-SNE plots showed that the NB patients were divided into two subgroups according to the SEQC clinical risk category (Figure 2e and Figure S3). Next, we investigated if patients grouped in different clinical outcome groups (i.e., high-risk, INSS stage, progression, MYCN amplification) presented different c-MYC/MYCN target-gene risk scores. We observed that all clinical groups with unfavorable outcome indicators exhibited significantly higher risk scores calculated using c-MYC and c-MYC ChIP, as well as MYCN targets (FDR < 0.05, Figures S4–S6). This suggests that the stratification based on c-MYC/MYCN target-gene risk scores performed well in NB patients.

**Figure 3:**
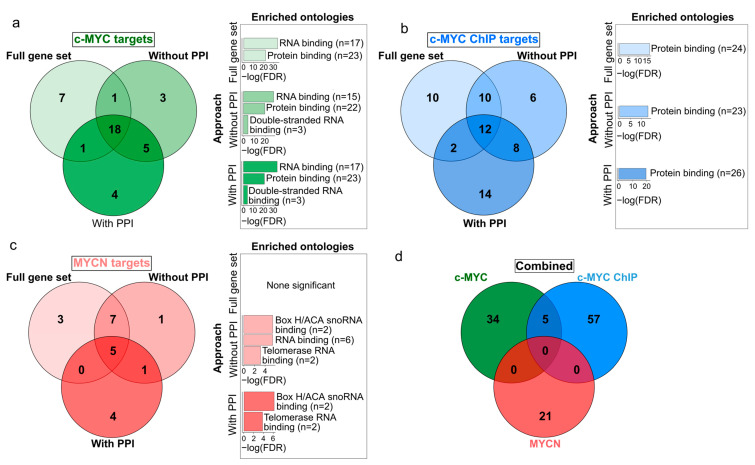
Venn diagrams displaying the differences and similarities in the c-MYC/MYCN target genes with prognostic value generated by different approaches. The different approaches to select the c-MYC/MCYN target genes (i.e., “Full gene set” (**a**), “Without PPI” (**b**), and “With PPI” (**c**) are detailed in Materials and Methods and displayed in Figure 1. (**d**) The combined diagram includes the merged c-MYC/MYCN target genes with prognostic value from each approach. Bar plots next to each gene set display significantly enriched gene ontologies with molecular function (Fisher’s exact test, one-tail, FDR < 0.05).

We further aimed at exploring the similarities between the set of c-MYC and MYCN target genes with prognostic power. While we obtained a relatively reduced number of c-MYC/MYCN targets using different approaches (Figure 3; Supplementary Table S3), we found that a large proportion of all these genes were retrieved by all the three approaches (46%, 19%, and 24% for c-MYC, c-MYC ChIP, and MYCN target genes, respectively, Figure 3a–c). Nevertheless, our analysis showed more significantly enriched ontology terms for the screened c-MYC/MYCN targets (i.e., “With PPI” and “Without PPI”) compared to the unfiltered gene sets (i.e., “Full gene set”, Figure 3a–c). This could be attributed to the fact that screen genes are more likely to have stronger biological functional connections and to share similar molecular mechanisms of action due to their involvement in the same pathways. Besides recovering this seemly cohesive function from the screened genes, we found no further evident advantage from pre-filtering the gene sets, particularly in terms of prognostic accuracy. In this way, for downstream analysis we favored the use of the unfiltered gene sets (i.e., “Full gene set”). The enrichment analysis of these prognostic genes showed that c-MYC targets were primarily involved in protein and RNA binding, with more than 80% and 60% of the c-MYC target genes annotated with each molecular function, respectively (Fisher Exact Test, one-tail, FDR < 0.05, Figure 3a). Meanwhile, MYCN targets were predominantly associated with snoRNA and telomerase RNA, with more than 14% and 10% of the MYCN target genes presenting each molecular function, respectively (Fisher Exact Test, one-tail, FDR < 0.05, Figure 3c). Interestingly, we discovered that c-MYC and MYCN targets with prognostic power represent distinct sets of genes (Figure 3d).

We revisited the analysis of gene sets to account for the influence of treatment protocols and therapy response on patient outcomes and risk levels. We adjusted the analysis for two groups of clinical variables: treatment response (“d_fav_all”) and a combination of treatment response, age at diagnosis, and gender. As with the previous results, we found a significant overlap in prognostic genes across different screening methods after accounting for the clinical variables (>40%, >27%, and >14% for c-MYC, c-MYC ChIP, and MYCN target genes, respectively). Furthermore, a sizable portion of these genes also appeared in the original unadjusted list, including *PLK1*, *ODC1*, and *CDK4* (Supplementary Table S3)

### 3.3. c-MYC/MYCN Prognostic Target Genes Are Direct Targets of c-MYC/MYCN in NB

**Figure 4:**
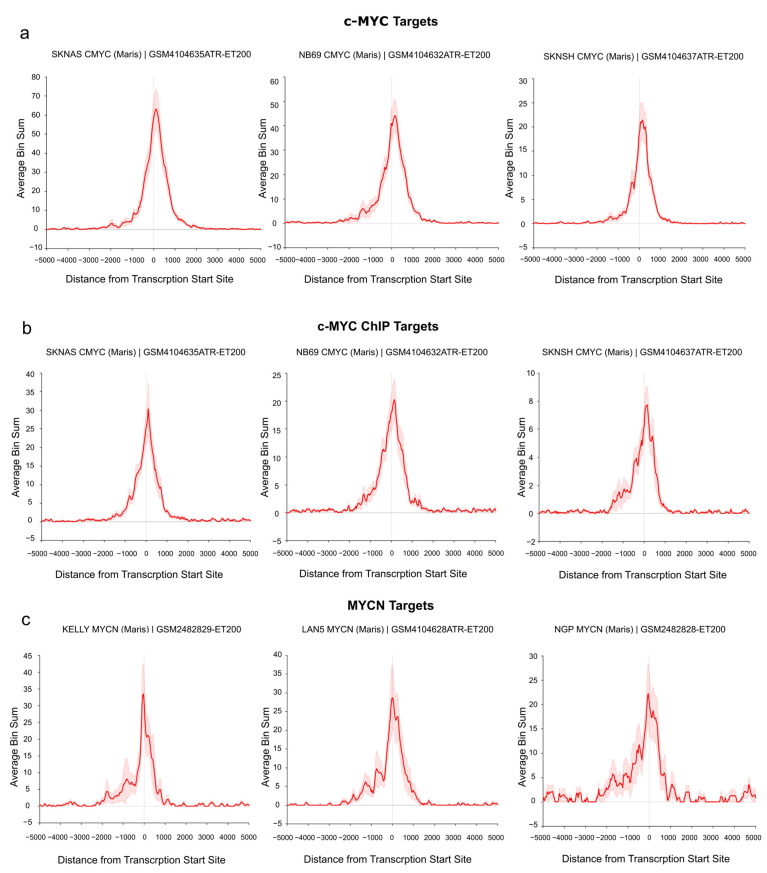
c-MYC/MYCN target genes with prognostic value are direct targets of c-MYC/MYCN in NB. The ChIP data illustrates the average enrichment of c-MYC around the transcription start sites (TSSs) of the c-MYC, c-MYC ChIP, and MYCN target genes shown in (**a**), (**b**), and (**c**), respectively. The target genes displayed here belong to the “Full gene set” approach. x-axis representation showed the distance measured from the TSS of all genes. y-axis represents the enrichment of c-MYC/MYCN ChIP-Seq peaks. The red solid line indicates the average enrichment of the ChIP-Seq peaks around TSS of all genes, while the red covered/hashed region represents the variation in ChIP-Seq peak enrichment among the c-MYC/MYCN target genes (“Full gene set”). The Figure was adapted from the R2 genome browser (http://r2.amc.nl, accessed on 15 August 2023), using the Maris et al. 2019 dataset [74] on the human genome assembly hg19.

To demonstrate the direct regulation of the identified target genes by c-MYC/MYCN, we investigated their binding at the transcription start site (TSS) of the “Full gene sets” using publicly available c-MYC/MYCN ChIP-Seq data. In particular, the analysis was performed on a set of human NB cell lines with and without *MYCN* amplification [74]. As shown in Figure 4a, we detected an enrichment of c-MYC ChIP-Seq peaks at the TSS of c-MYC target genes in three *MYCN*-non-amplified cell lines (i.e., SK-N-AS, NB69, and SK-N-SH, Figure 4a,b). Similarly, we found a remarkable number of MYCN ChIP-Seq peaks around the TSS of all MYCN targets in three *MYCN*-amplified cell lines (i.e., KELLY, LAN5, and NGP, Figure 4c). Additionally, a detailed examination showed an evident peak enrichment at the TSSs for selected c-MYC (i.e., *ODC1* [75] and *RAD50* [76] as well as MYCN targets (i.e., *DKC1* [77]) with relevance in NB (Figure S7).

### 3.4. Comparative Analysis of Patient Risk Stratification between c-MYC/MYCN Target Genes and Markers of Sympathoadrenal Development and NB

Neuroblastoma is considered to originate during the development of the sympathoadrenal system [3]. We studied the expression of c-MYC/MYCN target genes with prognostic power during development and in disease, focusing primarily on the target genes without applying any filtering criteria (i.e., “Full gene set”). In development, we found that c-MYC targets were significantly upregulated in murine sympathoblasts at E12.5 and E13.5, and in Schwann cell precursors (SCPs) at E12.5 (Fisher Exact Test, one-tail, FDR < 0.05, Figure S8a,b). A contrasting expression was observed for MYCN target genes, with a significant upregulation in sympathoblasts at E13.5 and in SCPs at E12.5 (Fisher Exact Test, one-tail, FDR < 0.05, Figure S8a,b). In NB, c-MYC target genes were significantly upregulated in neoplastic tumor clusters obtained from different datasets, while MYCN targets were significantly upregulated in tumor clusters from the 10x sequenced dataset (Fisher Exact Test, one-tail, FDR < 0.05, Figure S8c–e). These results indicate that while c-MYC and MYCN targets diverge in expression during development, they can be co-expressed in tumor cells.

**Figure 5:**
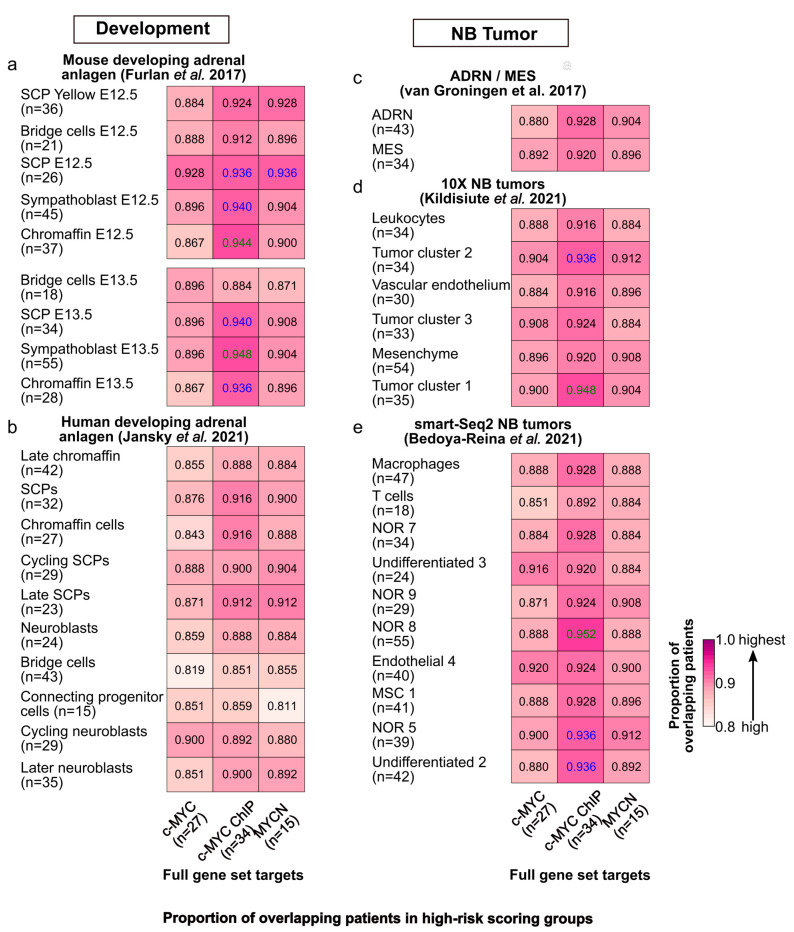
Similarities in risk-score classification of patients between c-MYC/MYCN target genes and genes expressed in different cell populations during sympathoadrenal development and in NB. Proportion (i.e., frequency) of patients classified in the group with the top median risk score by (1) c-MYC/MYCN target genes (full gene set, x-axis) and (2) markers of different cells clusters during (**a**) mouse [64] and (**b**) human [65] sympathoadrenal development, and (**c**–**e**) in NB [66,67,68]. The number of target genes/markers with prognostic value in each cluster is displayed in parentheses. The cells display in colors the pairs of gene sets for which the number of patients is significantly higher than expected from all the cases tested (Fisher’s exact test, one-tail, FDR < 0.05 in blue, and FDR < 0.01 in green).

We further compared the patient stratification capabilities of c-MYC/MYCN targets with prognostic power, with the marker genes of different cell clusters during murine and human sympathoadrenal development [64,65] as well as in NB [66,67,68]. To this end, these gene sets were independently modeled with LASSO-Cox in the same fashion as the c-MYC/MYCN targets. Our analysis demonstrated that significant proportions of patients (>80%, one-tailed, Fisher exact test, FDR < 0.05) were classified into the high-risk group by both genes expressed in different cell clusters during mouse adrenal anlagen development and by c-MYC/MYCN targets (Figure 5a). We observed similar results when considering c-MYC/MYCN targets and genes expressed in various cell clusters during human sympathoadrenal development (Figure 5b), and for different NB cell types/clusters in vitro (Figure 5c), as well as in vivo (Figure 5d,e). Nevertheless, this proportion was significantly higher between c-MYC direct targets (identified by ChIP) and developing murine adrenal anlagen that presented between 86% and 94% overlapping patients in the high-risk group (Fisher exact test, one-tail, FDR < 0.05). Similarly, the proportion of shared c-MYC/MYCN target genes and markers in development and disease with prognostic power was significantly high for murine sympathoadrenal development and NB comparisons, yet it was not in complete correlation with the results for patients (Fisher exact test, one-tail, FDR < 0.05, Figure S9a–e). One possible explanation for this is that the pool of genes screened by LASSO-Cox was larger for some datasets than for others. As a result, the probability of finding genes with high prognostic power increased for those datasets with more genes, so even if these gene sets are different, the patients are classified similarly into risk groups.

**Figure 6:**
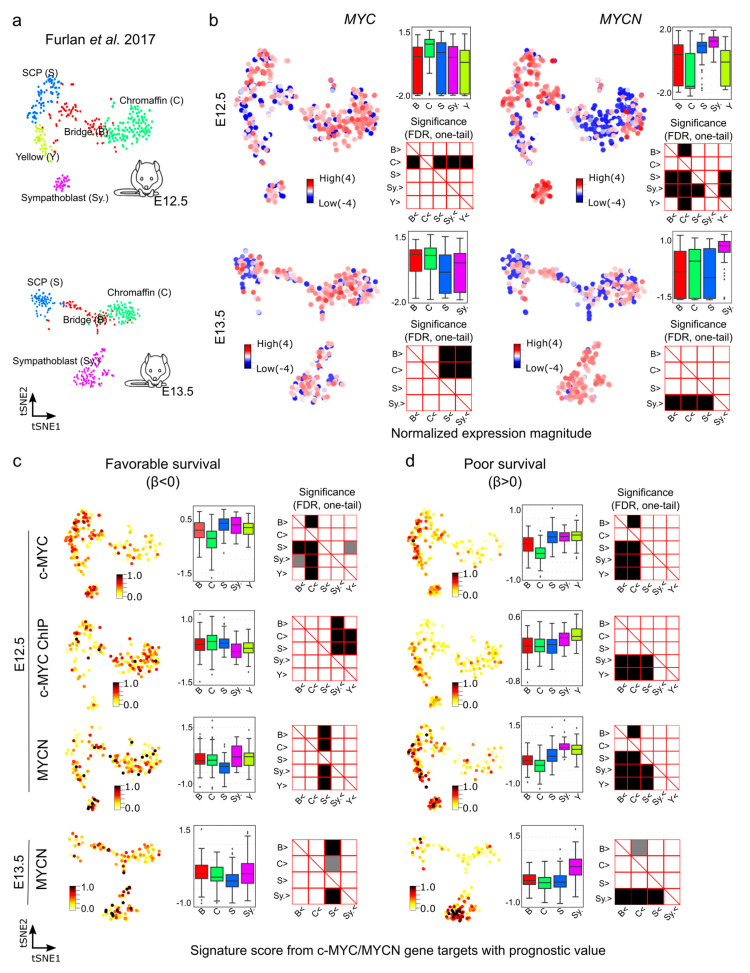
Signature scores of c-MYC/MYCN target genes with prognostic value during mouse sympathoadrenal development. (**a**) Illustration of the cell clusters within the developing sympathoadrenal anlagen at mouse embryonic stages E12.5 and E13.5. These clusters were investigated by Furlan et al. (2017) [64]. (**b**) *MYC* (encoding c-MYC) and *MYCN* expression patterns are displayed in single cells during development. Box plots illustrate the normalized expression magnitude in different cell clusters, and matrix plots the significance of pairwise comparisons between clusters, testing the hypothesis that clusters in the y-axis have a higher expression than clusters in the x-axis (Mann-Whitney U, one-tail, FDR). (**c**,**d**) Signature scores of c-MYC/MYCN target genes with favorable (β < 0) and poor (β > 0) prognostic value in cells of the murine developing sympathoadrenal anlagen at E12.5 and E13.5: B (bridge), C (chromaffin), S (SCPs), and Sy. (Sympathoblast). A higher signature score indicates a larger average expression of c-MYC/MYCN target genes than expected by chance. Box plots illustrate the distribution of signature scores for targets in single-cell clusters, and significance in pairwise comparisons are displayed in the adjunct matrix plots. Filled cells indicate that clusters in the y-axis present a significantly higher signature score than those in the x-axis (Mann-Whitney U test, one-tail, FDR). FDR values lower than 0.05 are displayed in grey, and lower than 0.01 in black.

We next aimed at further exploring the gene expression of c-MYC/MYCN targets with prognostic power during murine sympathoadrenal development. To this end, we obtained and examined their expression in the processed single cell data for stages E12.5 and E13.5 of the developing mouse adrenal anlagen [64]. At E12.5, *MYC* exhibits high expression, specifically in chromaffin cell clusters (Mann-Whitney U test, one-tail, FDR < 0.05, Figure 6a,b), while it is highly expressed in both chromaffin and bridge cells at E13.5 (Mann-Whitney U test, one-tail, FDR < 0.05, Figure S10). In contrast, at E12.5, *MYCN* shows high expression in SCPs and sympathoblast cells and remains high in sympathoblasts also at E13.5 (Mann-Whitney U test, one-tail, FDR < 0.05, Figure 6b). We computed the signature scores of the c-MYC/MYCN targets with a strong association with favorable (β < 0) or poor (β > 0) survivals in different gene clusters (Figure 6c,d). The signature score estimates the average expression of c-MYC/MYCN target genes compared to a set of random genes in each cell. We discovered that, overall, c-MYC and MYCN targets predicting poor survival were significantly expressed in sympathoblasts (Mann-Whitney U test, one-tail, FDR < 0.05) compared to chromaffin and bridge cells, irrespective of the developmental stage (i.e., E12.5 versus E13.5, Figure 6c,d), except for the c-MYC ChIP target, which presented no significance (Figure S10). Furthermore, MYCN targets predicting favorable survival showed lower expression in SCPs compared to other clusters (Mann-Whitney U test, one-tail, FDR < 0.05). For c-MYC targets predicting favorable survival, we obtained different results depending on the dataset, with the commonality that they were not significantly under-expressed in SCPs in comparison with other clusters (Mann-Whitney U test, one-tail, FDR < 0.05). This inconsistency may be attributed to the small fraction of favorable genes contributing to the list in each dataset (3/27, 9/34, and 5/15, respectively).

**Figure 7:**
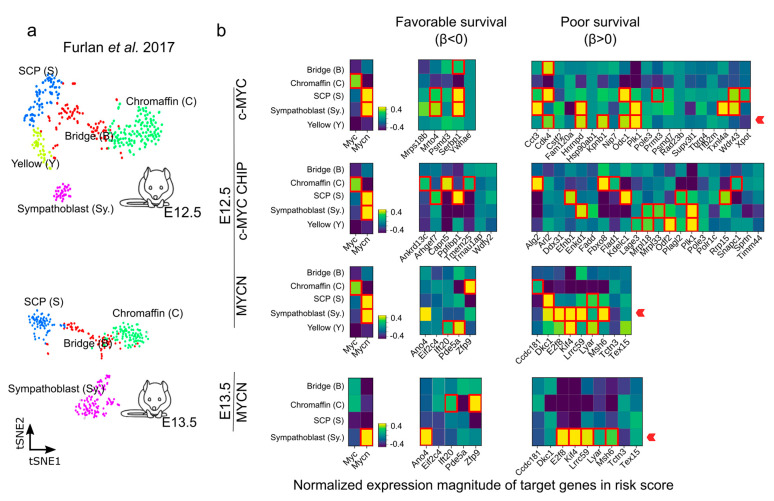
Expression of c-MYC/MYCN target genes with favorable (β < 0) and poor (β > 0) prognostic value during mouse sympathoadrenal development. (**a**,**b**) Average expression of *MYC* (encoding c-MYC), *MYCN,* and c-MYC/MYCN target genes with prognostic value were calculated for single-cell clusters during sympathoadrenal development [64]. The heatmaps illustrate the average normalized expression magnitude (computed by PAGODA [87]) for c-MYC/MYCN target genes with favorable (β < 0) and poor (β > 0) prognostic value during mouse sympathoadrenal development. Cells surrounded by a red square indicates genes significantly upregulated in cell clusters during development (Welch *t*-test, one-tail, FDR < 0.01). A red arrow signals the enrichment of target genes with favorable (β < 0) or poor (β > 0) prognostic value in cell clusters during development. The expression magnitude displayed is truncated to ranges between −0.4 and 0.4.

We further evaluated the genes with a strong association with favorable (β < 0) or poor (β > 0) survival during the murine sympathoadrenal development (Figure 7a,b and Figure S11). The expression of many 1:1 orthologues between human and mouse that are associated with a poor survival probability (β > 0) have been described to be important in NB outcome, including *DKC1*, *PLK1*, *FBXO8, ODC1*, and *CDK4* [43,75,77,78,79,80]. PLK1 is a serine/threonine kinase that plays a crucial role in cell division and cell cycle progression [81]. DKC1 and PLK1 stabilize MYC proteins, particularly MYCN, which in turn promotes their expression in a positive feedback loop. Furthermore, *DKC1* and *PLK1* upregulation is linked to unfavorable outcomes in NB [77,79]. Similarly, Ornithine decarboxylase (*ODC1*) is involved in polyamine biosynthesis and is upregulated in tumors, especially in MYC-driven cancers [82]. *ODC1* is a direct target of MYC proteins and is associated with poor survival in neuroblastoma [75,83]. CDK4 regulates cell cycle progression, and its inhibition in NB cells results in reduced growth by inducing G1 cell cycle arrest [78,84]. *ANO4*-encoding Anoctamin 4 is one example of a gene correlating with a favorable outcome and is a marker recently reported for the *zona glomerulosa* of the adrenal cortex [85], and its expression may be of relevance for calcium homeostasis and its role in cell differentiation and proliferation [86].

## 4. Discussion

Treatment options of NB patients depend on the combinations of several clinical and biological prognostic factors [88]. Despite the integration of multiple approaches such as conventional imaging (CT/MRI), MIBG, PET using FDG, bone marrow assessment, and histopathological classification, the current risk stratification for high-risk NB needs a more precise predictive ability. Notably, targeted therapy in NB now includes the consideration of *ALK* mutation status [89], whereby specific inhibitors are employed. In addition, targeted immune therapy such as anti-GD2 therapy is also utilized [90]. The incorporation of these customized treatment strategies has resulted in increased survival [89,90,91]. Nevertheless, treatments for subsets of high-risk NB patients need to be refined or individualized to reduce long-term side effects, such as omitting radiotherapy in selected cases based on predictive factors [92]. Furthermore, identifying additional prognostic target genes and biomarkers would improve the stratification of NB patients. Due to the genetic diversity observed in relapsed or refractory NB, targeting a single genetic marker is unlikely to result in a significant clinical response. Instead, a more promising approach would involve simultaneously targeting multiple key genetic or molecular markers using a combination of therapy modalities. With the goal of developing tools for the use of precision medicine in NB, previous studies have proposed several gene expression-based systems for risk stratification that can predict outcomes more accurately than traditional risk markers [18,93,94]. 

Here we hypothesized that a restricted group of c-MYC and MYCN target genes could contribute to better survival predictions of NB patients as compared to the expression of c-MYC or MYCN expression alone. In a seminal study exploring this possibility using a cohort of 88 patients, the authors suggested that MYCN targets are suitable for identifying patients with very poor outcomes [44]. We designed a framework that allowed us to select c-MYC/MYCN-targets with strong prognostic power and biomedical relevance, and to model a risk score that could stratify patients in different risk groups. To build a model, we used the well-known LASSO-Cox that allows for a selection of markers with strong predictive power (Figure 1). Our methodology demonstrated successful classification of patients into distinct survival outcome groups. 

Our methodology was aimed at selecting targets with both clinical relevance and notable protein-protein interaction (i.e., PPI). In particular, we aimed to use PPI screening to gain a deeper insight into the interactions between gene products, as a proxy to determine the pathways involved in NB onset and outcome. However, the accuracy of the prediction does not improve significantly when incorporating screened differentially expressed targets that possess clinical relevance and targets exhibiting remarkable interactions by PPI analysis (Figure 2). One possible explanation for this is the strong selection power of the LASSO-Cox model. Although this model has potential limitations, including the risks of overfitting and convergence issues, it is capable of selecting a small set of genes with robust predictive capabilities. This capability enables the model to consistently produce accurate results, even when handling extensive lists of genes as input. Despite these findings, the selection of c-MYC/MYCN target genes based on their clinical relevance and documented interaction demonstrated a higher degree of gene cohesiveness and biological relevance. This is supported by the larger enrichment and significance of gene ontologies (Figure 3). These results suggest that while pre-filtering may not significantly influence the resultant set of prognostic targets, the specific genes identified through meticulous analysis and the application of appropriate filters hold importance for future experimental investigations aimed at understanding NB.

As part of our experiments, we accounted for clinical variables that could significantly influence patient outcomes and risk categorization. To this end, we sacrificed some of the predictive power of the SEQC dataset, as only 272 patients had this clinical information. After making these adjustments, we observed that a substantial percentage of genes with prognostic relevance consistently emerged across different screening methods. Moreover, a notable fraction of these genes (ranging from 18% to 100%, with an average of 38%) also appeared in the unadjusted list, including *PLK1*, *ODC1*, and *CDK4*. While our results show, to a large extent, consistent findings, both with and without adjusting for clinical covariates, our analysis is far from ideal. We are using a variable (i.e., “d_fav_all”) as a proxy for response to therapy, which only partially captures the patients’ responses and is available for only half of them. Further studies could benefit from a more comprehensive record of variables related to the patients, particularly concerning their response to treatment.

We found that the predicted c-MYC targets with prognostic value differed from those of MYCN, independently of whether they were selected based on their clinical relevance and/or specific network interactions, or not. While the two genes are expected to be largely redundant, a previous study reported that c-MYC and MYCN share about 40% of their target genes using melanoma cells [95]. In NB, distinct targets of c-MYC and MYCN have been defined to have an impact on disease progression [38]. Nevertheless, the activation of c-MYC can drive approximately 10% of high-risk cases with MYCN-amplified clinical features [43]. The differences we observed in the c-MYC and MYCN targets could be arguably associated with the cellular context in which c-MYC and MYCN act, or be due to differences in their function [38,96]. Disentangling the regulatory relevance of context and function in development and malignancy is not trivial. For example, our results indicate that MYCN binds with a high affinity to the promoter of *DKC1* in Kelly and NGP cells. Notably, *DKC1* expression has recently been shown to be higher in *MYCN*-amplified cell lines and primary NB tumors when compared to *MYCN*-non-amplified NBs [97]. Even so, from a time-wise perspective, *DKC1* has been shown to be activated consecutively by c-MYC and then by MYCN after promoting its expression in the SH-EP MYCN cell line [38]. This suggests that the transcription of some targets might be promoted with different strengths by the activities of c-MYC and MYCN in both a cell- and time-dependent context (Figure 4). Furthermore, *MYCN* and *MYC* expression are mutually exclusive in NB cells after they form a self-regulatory loop [38,98]. 

Remarkably, even if the targets associated with poor outcome are different for c-MYC and MYCN, they are significantly up-regulated in murine sympathoblasts, indicating that a shared mechanism or a similar cell context (i.e., microenvironment during development) can promote a more aggressive form of the malignancy. In particular, hypoxia is one microenvironment factor that could have relevance for both sympathoadrenal development and the malignancy onset and evolution [99]. The oxygen-sensing HIF proteins could interact with both MYC and c-MYC, and finely regulate their activity during development. As consequence, the miss-regulation of the expression and/or activity of HIF proteins could have a role in NB onset and aggressiveness. Other microenvironment factors of relevance include cells in the tumor surroundings, such as immune cells, that could contribute to an immune-suppressive environment, as previously suggested for glioblastoma [100].

We observed that the classification of patients into risk groups by the computed median risk score was high for all cases but was highest for the predictions obtained from genes expressed in cell clusters of the mouse developing adrenal gland (Figure 5 and Figure 6). In particular, the proportion of predicted patients with worse outcomes was significantly more similar between c-MYC targets obtained by ChIP and the developing murine adrenal anlagen. This is most likely due to the large list of genes that both studies included, from which LASSO-Cox could select powerful markers. Notably, c-MYC and MYCN target genes present a different expression pattern in the developing sympathoadrenal system. c-MYC and MYCN targets predicting worse outcomes were more likely expressed in sympathoblasts, while MYCN targets predicting better outcomes were less expressed in SCPs than in other cell clusters. Research conducted in zebrafish has shown that the abnormal overexpression of *MYCN* in sympathoadrenal progenitor cells inhibits the maturation of chromaffin cells, leading to NB formation [101]. This is consistent with our results indicating that MYCN targets with good prognosis exhibit low expression in chromaffin cell clusters at mouse stages E12.5 and E13.5. Interestingly, a dramatic reduction of *Mycn* expression in SCPs was observed between mouse E12.5 and E13.5. It is possible that c-MYC and MYCN play overlapping roles during development. However, as mid-gestation approaches, it is likely that they start to function in a tissue-specific manner [20].

The NB cell of origin is considered to either be a developing and incompletely committed precursor cell or an undifferentiated but already committed cell from neural-crest tissues [3,102]. *MYCN* is highly expressed in the early post-migratory neural crest and regulates migration and growth of cells during normal murine sympathoadrenal development into neuronal and chromaffin cells [7]. The enrichment of *MYCN*-target genes in sympathoblasts but not in chromaffin or bridge cells suggests that MYCN activity, based on MYCN targe-gene expression, might be blocking normal murine sympathoadrenal development and/or pushing the cells towards a sympathoblast fate. *MYCN* expression is induced in the sympathetic ganglia during gestation and then switched off before birth, the time when *MYC* expression is turned on [103]. Notably, we found that c-MYC targets predicting poor outcome were also expressed in sympathoblasts but not in chromaffin or bridge cells. This suggests that high c-MYC activity may have a similar effect as MYCN in blocking the maturation of sympathetic ganglia. Recent studies have suggested that the transcriptome of some human NB cells resemble that of fetal sympathoblasts, revealing their early developmental stage and potential as targets for further investigation for therapeutic approaches [67,68]. Our findings align with a recent study indicating that tumors characterized by a higher proportion of neuroblast-like cells exhibit a poorer prognosis [65]. Nevertheless, our results are limited to targets of MYCN and c-MYC during development, and we have thus not addressed the relevance of other post-natal sympathoadrenal cell populations and/or molecular mechanisms resulting in poor outcome in NB (i.e., cell response to DNA damage, DNA repair, RNA splicing, and cell movement) [68]. 

The high expression of c-MYC/MYCN target genes indicative of poor prognostic value in developing sympathoblasts suggests that targeting NB such as this cell population may present a novel treatment strategy for treating some of the worst outcome cases. Considering the challenges associated with directly targeting c-MYC and MYCN, a viable alternative approach could be to target downstream components [104]. In our results, well known druggable c-MYC and MYCN target genes, indicating poor prognosis and expressed in sympathoblast, include *PLK1*, and other potentially druggable target genes include *ODC1* and *CDK4*. PLK1 inhibition with Volasertib (i.e., BI6727) results in tumor growth suppression in NB xenografts [81]. Similarly, ODC1 inhibition by difluoromethylornithine (DFMO) has shown promising results, both in vitro and in vivo, and it is being evaluated in previous phase II clinical trials for NB patients [105]. Finally, CDK4 can be inhibited by ribociclib or abemaciclib. Both have demonstrated the effective reduction of proliferation in a wide range of NB cell lines [84,106]. 

We have focused on studying c-MYC and MYCN targets with prognostic value. Furthermore, several other genes, such as *TERT*, *ATRX*, and *NTRK2*, are also associated with poor outcomes. In this regard, a recent study conducted a review of prognostic genetic markers in NB, highlighting a lack of uniformity across various publications [107]. While the diverse studies assessed in the article utilized different methodologies and served different purposes, it is not surprising that three target genes with prognostic value from our study, namely *ARHGEF7*, *SNAPC1*, and *ODC1*, were also identified as having prognostic significance in two of the publications reviewed ([108] *n* = 42, [109] *n* = 52). Ultimately, various factors related to the clinical heterogeneity of NB may explain differences in the prognostic markers observed in different studies. First, pretreatment prior to surgery can vary among cohorts depending on the treatment protocol, evolving over time. Second, the location or site of the analyzed tissue could impact the predictive ability of the genes, as there is considerable intra-patient genetic heterogeneity, which may confound results such as expression in association with survival. Furthermore, the rationale for selecting distinct gene sets for marker screening depends on the specific objectives of the study. Our objective is to expand the knowledge and predictive ability of c-MYC and MYCN and their target genes. In our study, our ultimately aim was targeting the cell of origin of the malignancy for early diagnosis and treatment. 

By identifying distinct molecular subtypes and their prognostic value, our approach holds the potential to inform tailored therapeutic interventions, leading to improved patient outcomes for those classified as high risk. In particular, we found druggable protein targets from genes predicting poor outcome (as detailed above). These genes are expressed during development, arguably in the timeframe in which the initial oncogenic event took place in NB [110]. While targeting c-MYC and/or MYCN for cancer treatment is a major challenge, these proteins represent a potential group of targets for which drugs are already available or are in development. Nevertheless, further validation studies in larger cohorts and clinical trials to fully realize the translational and therapeutic role of our approach are required.

## 5. Conclusions

In conclusion, our findings shed new light on the significance of c-MYC/MYCN target genes in NB tumorigenesis and their prognostic value in stratifying patients. These data add valuable dimensions to the existing literature by providing a comprehensive understanding of the molecular mechanisms underlying NB development and offering a robust framework for risk classification by utilizing a panel of target genes rather than one single biomarker such as c-MYC or MYCN alone. The comparison of c-MYC/MYCN targets with markers of sympathoadrenal development and NB underscored their robust patient stratification capabilities. Insights into the expression of these genes during various developmental stages and in disease contexts further enrich our understanding, unveiling the intricate interplay between gene expression patterns, clinical outcomes, and developmental stages in NB. We anticipate that these findings will pave the way for future investigations, thereby driving the development of targeted therapies and personalized treatment approaches for NB patients. 

## Data Availability

The study’s original contributions are in the article/Supplementary Materials, and further inquiries can be directed to the corresponding author.

## Supplementary Materials

The following supporting information can be downloaded at: https://www.mdpi.com/article/10.3390/cancers15184599/s1, Figure S1: Validation of the prognostic performance of risk-score models in the Kocak and Versteeg cohorts; Figure S2: Kaplan-Meier curves illustrate the predictive ability of the risk-score models for different targets in the SEQC, Kocak and Versteeg cohorts; Figure S3: t-SNE illustration of the transcriptional similarities between patients for genes with prognostic value; Figure S4: Violin-/boxplots of the risk-scores computed with the “Full gene set” of c-MYC/MYCN targets on patient groups with different clinical variables; Figure S5: Violin-/boxplots of the risk-scores computed for c-MYC/MYCN targets filtered with the “Without PPI” approach on patient groups with different clinical variables; Figure S6: Violin-/boxplots of the risk-scores computed for c-MYC/MYCN targets filtered with the “With PPI” approach on groups of patients with different clinical variables; Figure S7: c-MYC/MYCN directly binds to the transcription start site (TSS) of c-MYC target genes, c-MYC ChIP target genes, and MYCN target genes; Figure S8: Overlapping proportion of c-MYC/MYCN target genes (obtained from the SEQC dataset) and markers for different cell clusters during sympathoadrenal development and in NB; Figure S9: Overlapping proportion of c-MYC/MYCN target genes (obtained from the SEQC dataset) and markers with predictive power for different cell clusters during sympathoadrenal development and in NB; Figure S10: Signature scores of c-MYC/MYCN targets with prognostic value during mouse sympathoadrenal development at E13.5; Figure S11: Expression of c-MYC/MYCN targets with favorable (β < 0) and poor (β > 0) prognostic value during mouse sympathoadrenal development at E13.5; Table S1: Unfiltered c-MYC/MYCN target sets included for the analysis; Table S2: Target genes before LASSO-Cox screening; Table S3: Target genes from different approaches.
