# Supplementary material for: Target Genes of c-MYC and MYCN with Prognostic Power in Neuroblastoma Exhibit Different Expressions during Sympathoadrenal Development
Source: Cancers (Basel). 2023 Sep 16;15(18):4599. doi: 10.3390/cancers15184599 (PMC10527308; doi:10.3390/cancers15184599)
Supplement: Supplementary file 1 [file cancers-15-04599-s001.zip › Fig S1-11.pdf]

Figure S1

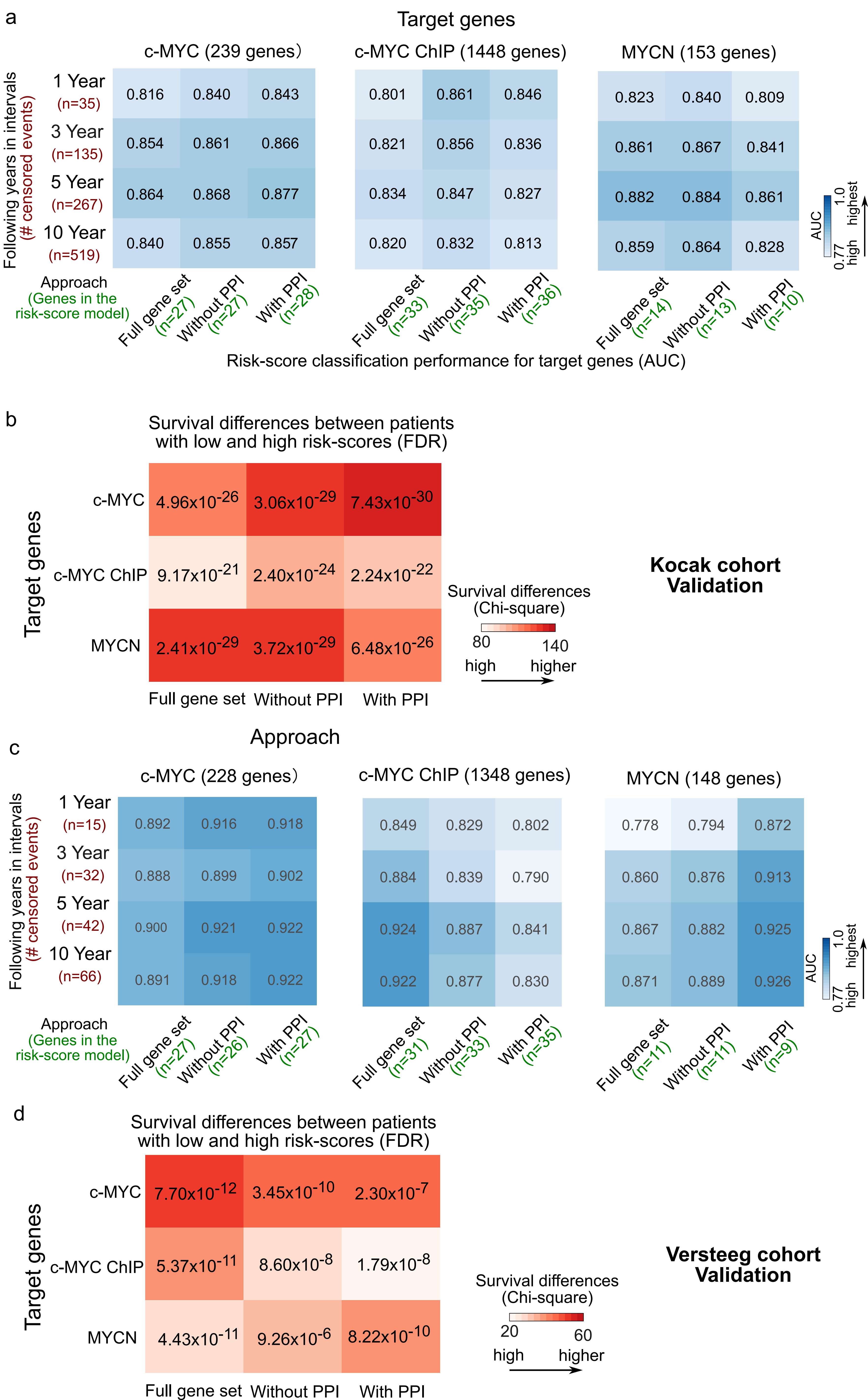
a
Target genes
c-MYC (239 genes)
c-MYC ChIP (1448 genes)
MYCN (153 genes)
Following years in intervals
(# censored events)
1 Year
(n=35)
3 Year
(n=135)
5 Year
(n=267)
10 Year
(n=519)
0.816 0.840 0.843
0.854 0.861 0.866
0.864 0.868 0.877
0.840 0.855 0.857
0.801 0.861 0.846
0.821 0.856 0.836
0.834 0.847 0.827
0.820 0.832 0.813
0.823 0.840 0.809
0.861 0.867 0.841
0.882 0.884 0.861
0.859 0.864 0.828
AUC
1.0
highest
0.77
high
Approach
(Genes in the
risk-score model)
Full gene set (n=27)
Without PPI (n=27)
With PPI (n=28)
Full gene set (n=33)
Without PPI (n=35)
With PPI (n=36)
Full gene set (n=14)
Without PPI (n=13)
With PPI (n=10)
Risk-score classification performance for target genes (AUC)
b
Survival differences between patients
with low and high risk-scores (FDR)
Target genes
c-MYC
c-MYC ChIP
MYCN
4.96x10-26 3.06x10-29 7.43x10-30
9.17x10-21 2.40x10-24 2.24x10-22
2.41x10-29 3.72x10-29 6.48x10-26
Full gene set
Without PPI
With PPI
Approach
Survival differences
(Chi-square)
80
140
high
higher
Kocak cohort
Validation
c
c-MYC (228 genes)
c-MYC ChIP (1348 genes)
MYCN (148 genes)
Following years in intervals
(# censored events)
1 Year
(n=15)
3 Year
(n=32)
5 Year
(n=42)
10 Year
(n=66)
0.892 0.916 0.918
0.888 0.899 0.902
0.900 0.921 0.922
0.891 0.918 0.922
0.849 0.829 0.802
0.884 0.839 0.790
0.924 0.887 0.841
0.922 0.877 0.830
0.778 0.794 0.872
0.860 0.876 0.913
0.867 0.882 0.925
0.871 0.889 0.926
AUC
1.0
highest
0.77
high
Approach
(Genes in the
risk-score model)
Full gene set (n=27)
Without PPI (n=26)
With PPI (n=27)
Full gene set (n=31)
Without PPI (n=33)
With PPI (n=35)
Full gene set (n=11)
Without PPI (n=11)
With PPI (n=9)
d
Survival differences between patients
with low and high risk-scores (FDR)
Target genes
c-MYC
c-MYC ChIP
MYCN
7.70x10-12 3.45x10-10 2.30x10-7
5.37x10-11 8.60x10-8 1.79x10-8
4.43x10-11 9.26x10-6 8.22x10-10
Full gene set
Without PPI
With PPI
Survival differences
(Chi-square)
20
60
high
higher
Versteeg cohort
Validation

Figure S2

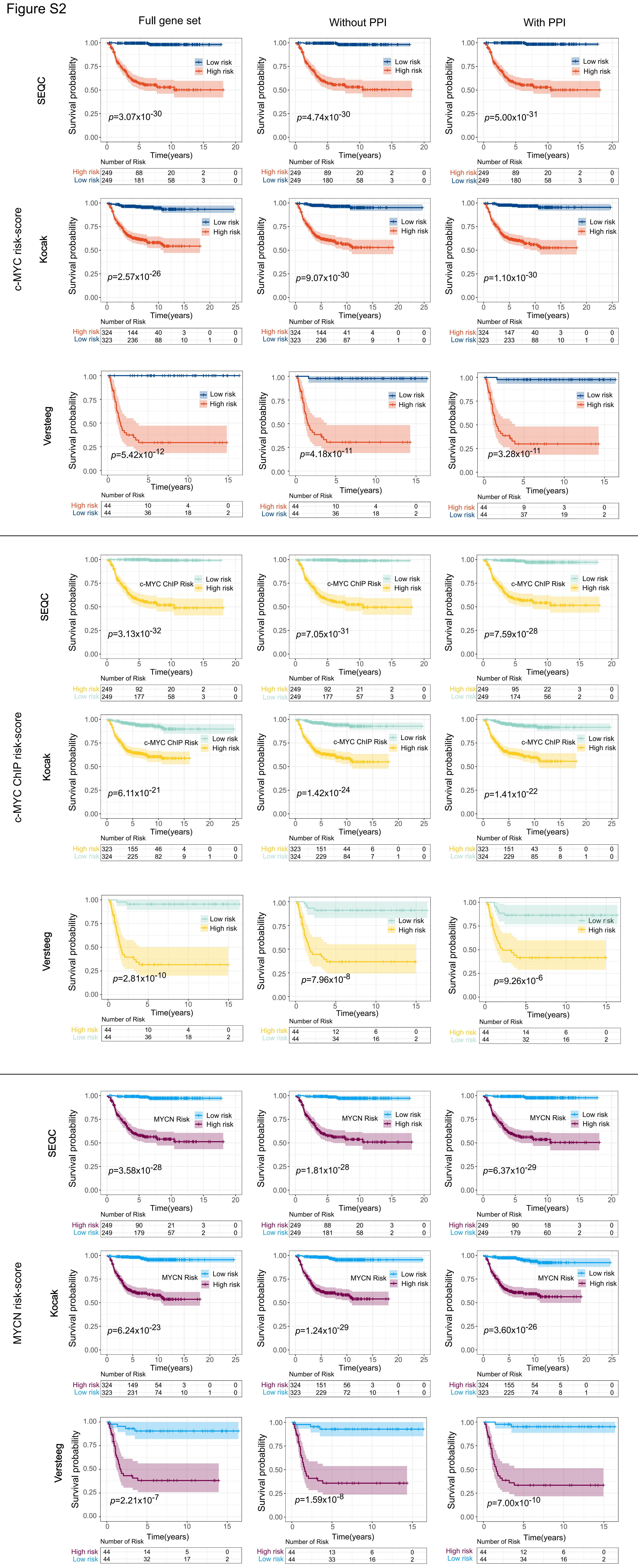

Full gene set
Without PPI
With PPI
c-MYC risk-score
c-MYC ChIP risk-score
MYCN risk-score
SEQC
Kocak
Versteeg
Survival probability
Time(years)
Number of Risk
Low risk
High risk
c-MYC ChIP Risk
MYCN Risk
p=3.07x10^-30
p=4.74x10^-30
p=5.00x10^-31
p=2.57x10^-26
p=9.07x10^-30
p=1.10x10^-30
p=5.42x10^-12
p=4.18x10^-11
p=3.28x10^-11
p=3.13x10^-32
p=7.05x10^-31
p=7.59x10^-28
p=6.11x10^-21
p=1.42x10^-24
p=1.41x10^-22
p=2.81x10^-10
p=7.96x10^-8
p=9.26x10^-6
p=3.58x10^-28
p=1.81x10^-28
p=6.37x10^-29
p=6.24x10^-23
p=1.24x10^-29
p=3.60x10^-26
p=2.21x10^-7
p=1.59x10^-8
p=7.00x10^-10
High risk 249 88 20 2 0
Low risk 249 181 58 3 0
High risk 249 89 20 2 0
Low risk 249 180 58 3 0
High risk 249 89 20 2 0
Low risk 249 180 58 3 0
High risk 324 144 40 3 0 0
Low risk 323 236 88 10 1 0
High risk 324 144 41 4 0 0
Low risk 323 236 87 9 1 0
High risk 324 147 40 3 0 0
Low risk 323 233 88 10 1 0
High risk 44 10 4 0
Low risk 44 36 18 2
High risk 44 10 4 0
Low risk 44 36 18 2
High risk 44 9 3 0
Low risk 44 37 19 2
High risk 249 92 20 2 0
Low risk 249 177 58 3 0
High risk 249 92 21 2 0
Low risk 249 177 57 3 0
High risk 249 95 22 3 0
Low risk 249 174 56 2 0
High risk 323 155 46 4 0 0
Low risk 324 225 82 9 1 0
High risk 323 151 44 6 0 0
Low risk 324 229 84 7 1 0
High risk 323 151 43 5 0 0
Low risk 324 229 85 8 1 0
High risk 44 10 4 0
Low risk 44 36 18 2
High risk 44 12 6 0
Low risk 44 34 16 2
High risk 44 14 6 0
Low risk 44 32 16 2
High risk 249 90 21 3 0
Low risk 249 179 57 2 0
High risk 249 88 20 2 0
Low risk 249 181 58 3 0
High risk 249 90 18 3 0
Low risk 249 179 60 2 0
High risk 324 149 54 3 0 0
Low risk 323 231 74 10 1 0
High risk 324 151 56 3 0 0
Low risk 323 229 72 10 1 0
High risk 324 155 54 5 0 0
Low risk 323 225 74 8 1 0
High risk 44 14 5 0
Low risk 44 32 17 2
High risk 44 13 6 0
Low risk 44 33 16 2
High risk 44 12 6 0
Low risk 44 34 16 2

Figure S3

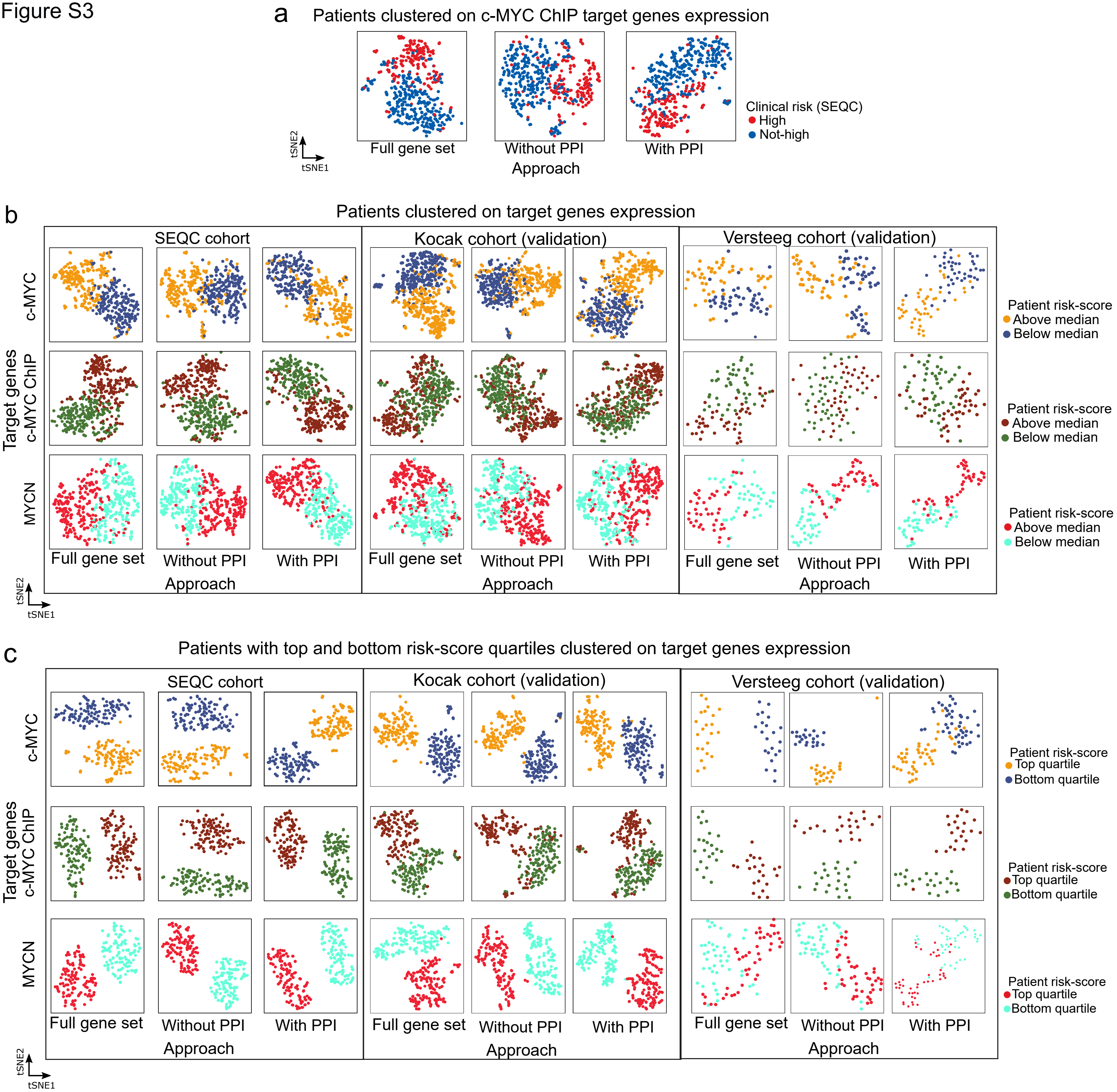
a
Patients clustered on c-MYC ChIP target genes expression
Full gene set
Without PPI
With PPI
Approach
tSNE2
tSNE1
Clinical risk (SEQC)
High
Not-high
b
Patients clustered on target genes expression
SEQC cohort
Kocak cohort (validation)
Versteeg cohort (validation)
Target genes
c-MYC
c-MYC ChIP
MYCN
Full gene set
Without PPI
With PPI
Approach
Patient risk-score
Above median
Below median
Patient risk-score
Above median
Below median
Patient risk-score
Above median
Below median
c
Patients with top and bottom risk-score quartiles clustered on target genes expression
SEQC cohort
Kocak cohort (validation)
Versteeg cohort (validation)
Target genes
c-MYC
c-MYC ChIP
MYCN
Full gene set
Without PPI
With PPI
Approach
Patient risk-score
Top quartile
Bottom quartile
Patient risk-score
Top quartile
Bottom quartile
Patient risk-score
Top quartile
Bottom quartile

Figure S4

Full gene set

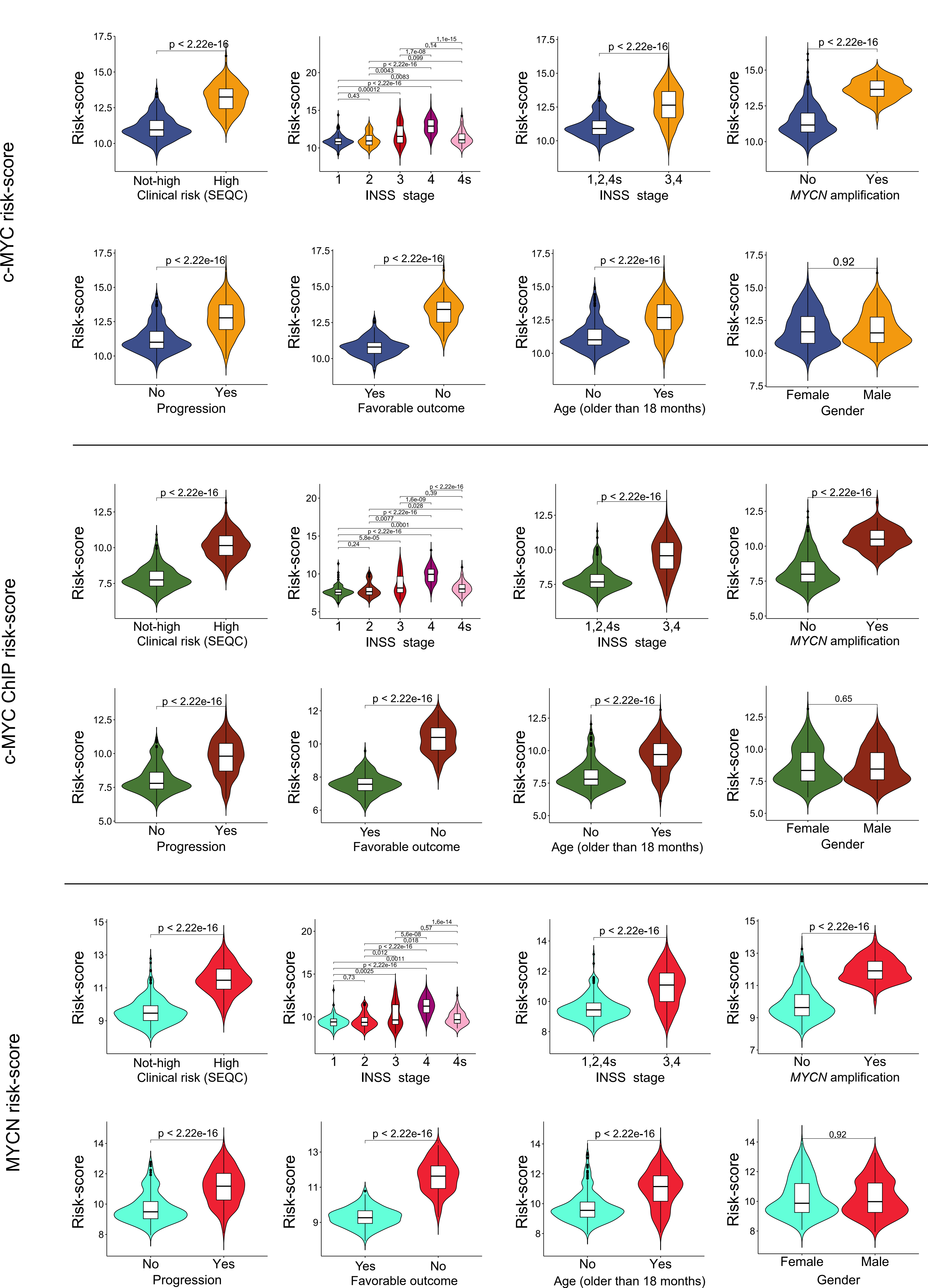

Figure S5

Without PPI

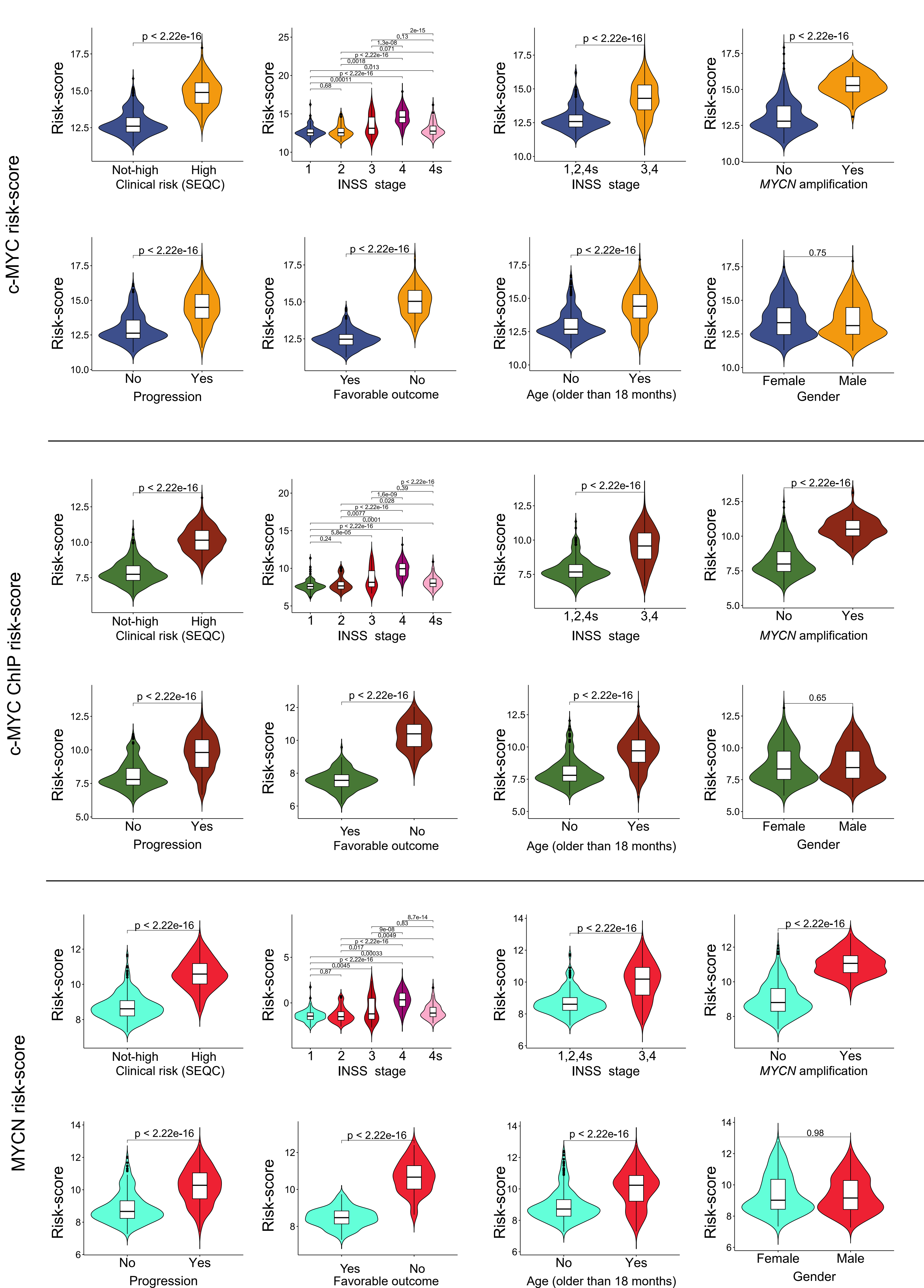

Figure S6

With PPI

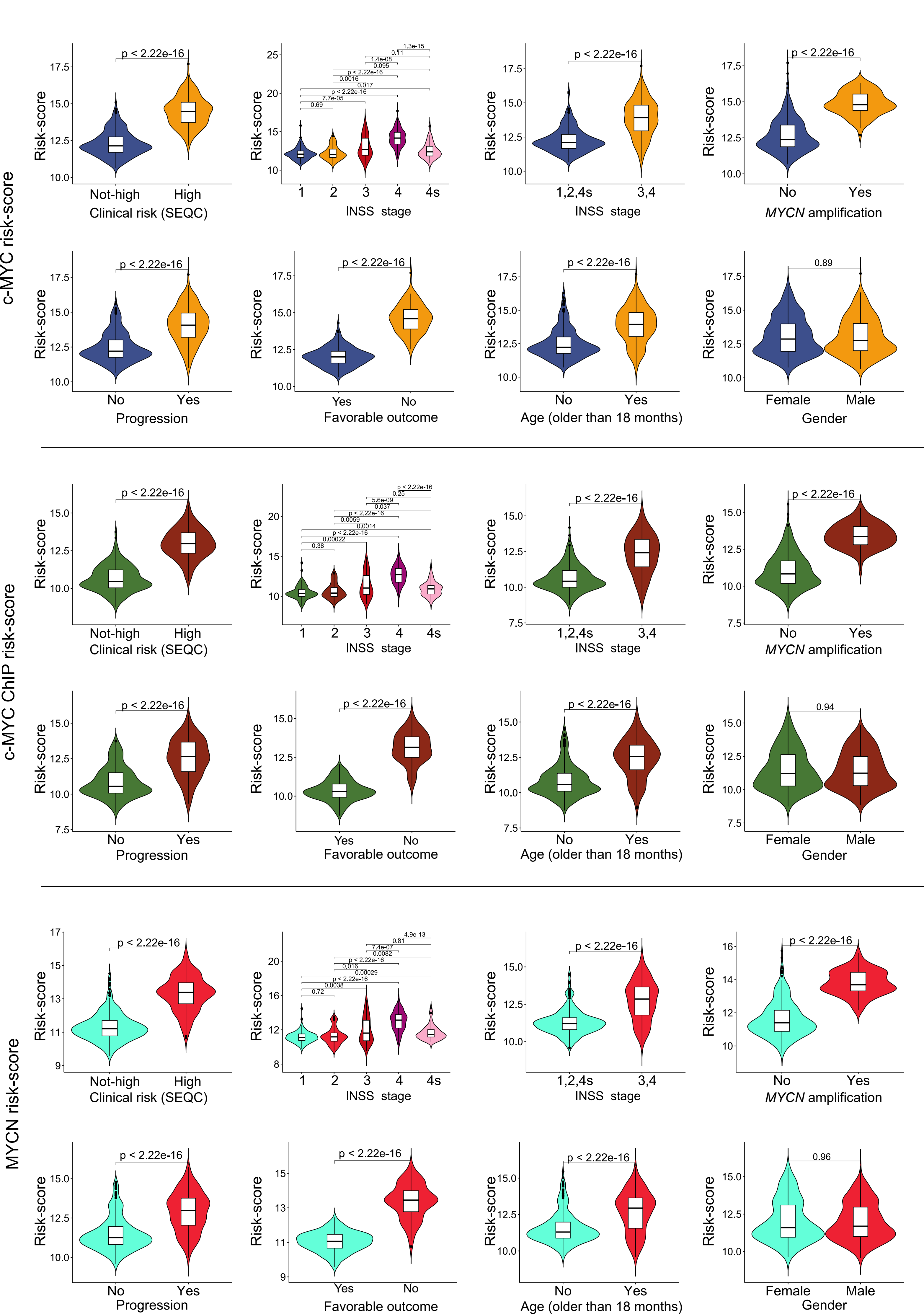

Figure S7

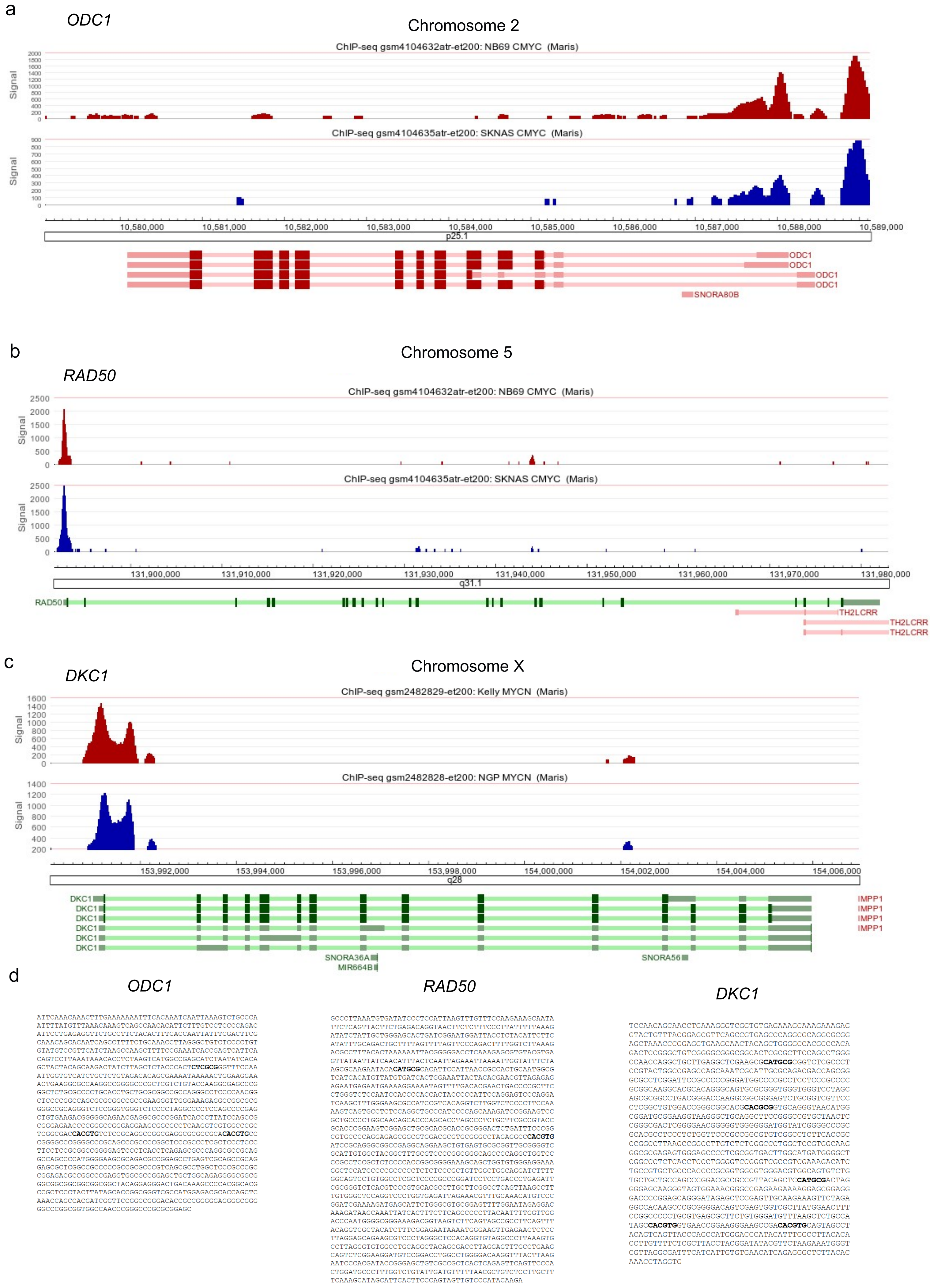

Adapted from R2 (http://r2.amc.nl)

Figure S8

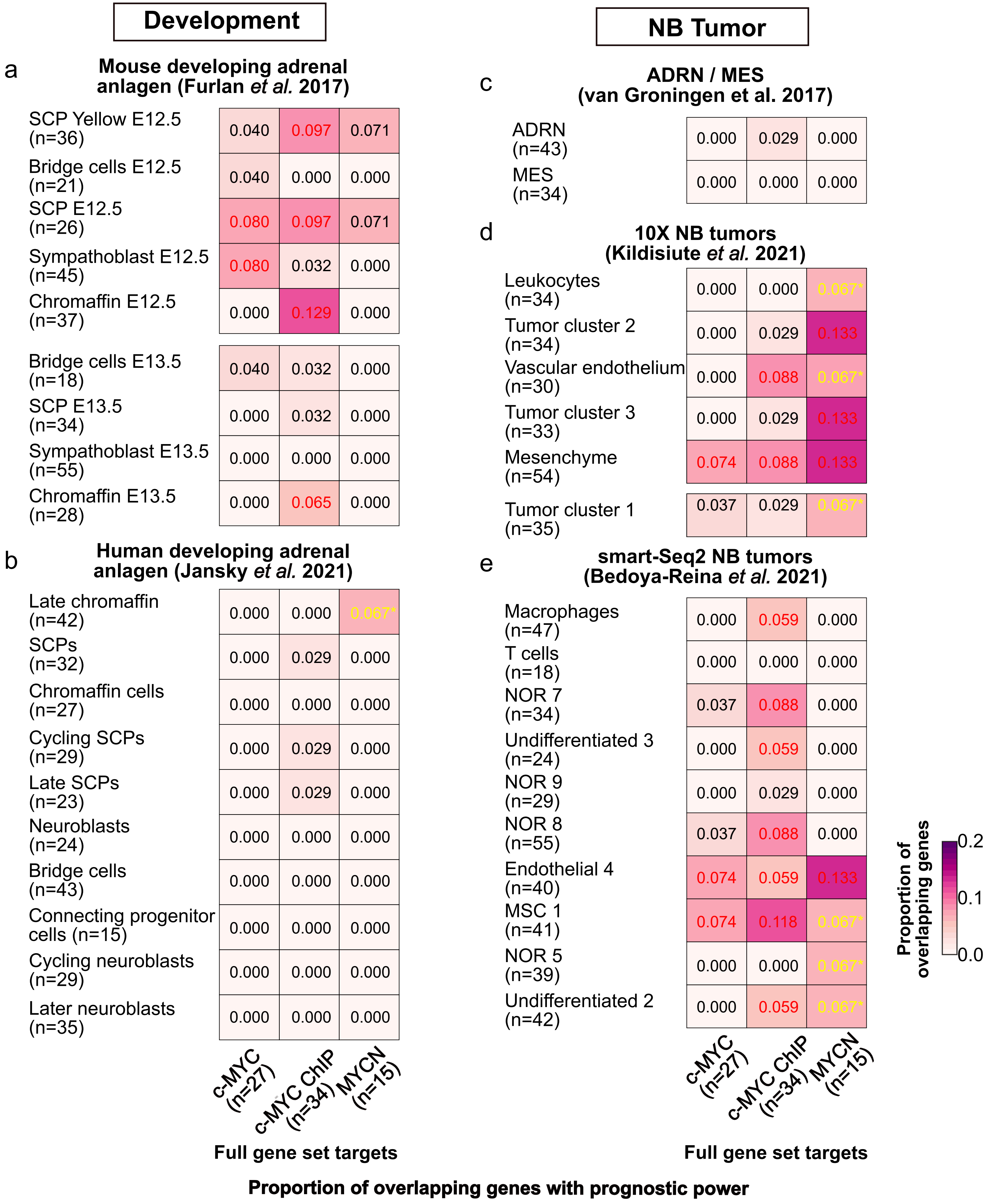
Development
a
Mouse developing adrenal anlagen (Furlan et al. 2017)
SCP Yellow E12.5 (n=36) 0.040 0.097 0.071
Bridge cells E12.5 (n=21) 0.040 0.000 0.000
SCP E12.5 (n=26) 0.080 0.097 0.071
Sympathoblast E12.5 (n=45) 0.080 0.032 0.000
Chromaffin E12.5 (n=37) 0.000 0.129 0.000
Bridge cells E13.5 (n=18) 0.040 0.032 0.000
SCP E13.5 (n=34) 0.000 0.032 0.000
Sympathoblast E13.5 (n=55) 0.000 0.000 0.000
Chromaffin E13.5 (n=28) 0.000 0.065 0.000
b
Human developing adrenal anlagen (Jansky et al. 2021)
Late chromaffin (n=42) 0.000 0.000 0.067*
SCPs (n=32) 0.000 0.029 0.000
Chromaffin cells (n=27) 0.000 0.000 0.000
Cycling SCPs (n=29) 0.000 0.029 0.000
Late SCPs (n=23) 0.000 0.029 0.000
Neuroblasts (n=24) 0.000 0.000 0.000
Bridge cells (n=43) 0.000 0.000 0.000
Connecting progenitor cells (n=15) 0.000 0.000 0.000
Cycling neuroblasts (n=29) 0.000 0.000 0.000
Later neuroblasts (n=35) 0.000 0.000 0.000
c-MYC (n=27)
c-MYC ChIP (n=34)
MYCN (n=15)
Full gene set targets
NB Tumor
c
ADRN / MES (van Groningen et al. 2017)
ADRN (n=43) 0.000 0.029 0.000
MES (n=34) 0.000 0.000 0.000
d
10X NB tumors (Kildisiute et al. 2021)
Leukocytes (n=34) 0.000 0.000 0.067*
Tumor cluster 2 (n=34) 0.000 0.029 0.133
Vascular endothelium (n=30) 0.000 0.088 0.067*
Tumor cluster 3 (n=33) 0.000 0.029 0.133
Mesenchyme (n=54) 0.074 0.088 0.133
Tumor cluster 1 (n=35) 0.037 0.029 0.067*
e
smart-Seq2 NB tumors (Bedoya-Reina et al. 2021)
Macrophages (n=47) 0.000 0.059 0.000
T cells (n=18) 0.000 0.000 0.000
NOR 7 (n=34) 0.037 0.088 0.000
Undifferentiated 3 (n=24) 0.000 0.059 0.000
NOR 9 (n=29) 0.000 0.029 0.000
NOR 8 (n=55) 0.037 0.088 0.000
Endothelial 4 (n=40) 0.074 0.059 0.133
MSC 1 (n=41) 0.074 0.118 0.067*
NOR 5 (n=39) 0.000 0.000 0.067*
Undifferentiated 2 (n=42) 0.000 0.059 0.067*
c-MYC (n=27)
c-MYC ChIP (n=34)
MYCN (n=15)
Full gene set targets
Proportion of overlapping genes
0.2
0.1
0.0
Proportion of overlapping genes with prognostic power

Figure S9

Development

a — Mouse developing adrenal anlagen (Furlan *et al.* 2017)

	c-MYC (n=27)	c-MYC ChIP (n=34)	MYCN (n=15)
SCP Yellow E12.5 (n=643)	0.200	0.129	0.286
Bridge cells E12.5 (n=475)	0.080	0.000	0.000
SCP E12.5 (n=1704)	0.320	0.194	0.143
Sympathoblast E12.5 (n=1241)	0.280	0.129	0.286
Chromaffin E12.5 (n=2210)	0.000	0.226	0.143
Bridge cells E13.5 (n=615)	0.040	0.032	0.000
SCP E13.5 (n=1488)	0.080	0.097	0.000
Sympathoblast E13.5 (n=1839)	0.360	0.097	0.429
Chromaffin E13.5 (n=2037)	0.040	0.194	0.143

b — Human developing adrenal anlagen (Jansky *et al.* 2021)

	c-MYC (n=27)	c-MYC ChIP (n=34)	MYCN (n=15)
Late chromaffin (n=118)	0.000	0.000	0.067
SCPs (n=317)	0.000	0.029	0.000
Chromaffin cells (n=253)	0.000	0.000	0.000
Cycling SCPs (n=371)	0.000	0.029	0.067
Late SCPs (n=252)	0.000	0.029	0.000
Neuroblasts (n=87)	0.000	0.000	0.000
Bridge cells (n=80)	0.000	0.000	0.000
Connecting progenitor cells (n=52)	0.000	0.000	0.000
Cycling neuroblasts (n=130)	0.000	0.000	0.067
Later neuroblasts (n=137)	0.000	0.000	0.000

Full gene set targets

NB Tumor

c — ADRN / MES (van Groningen et al. 2017)

	c-MYC (n=27)	c-MYC ChIP (n=34)	MYCN (n=15)
ADRN (n=367)	0.000	0.059	0.067
MES (n=484)	0.037	0.000	0.000

d — 10X NB tumors (Kildisiute *et al.* 2021)

	c-MYC (n=27)	c-MYC ChIP (n=34)	MYCN (n=15)
Leukocytes (n=1567)	0.000	0.000	0.067
Tumor cluster 2 (n=8179)	0.778	0.618	0.667
Vascular endothelium (n=1149)	0.000	0.088	0.067
Tumor cluster 3 (n=8954)	0.815	0.559	0.600
Mesenchyme (n=3686)	0.259	0.147	0.333
Tumor cluster 1 (n=9465)	0.815	0.618	0.600

e — smart-Seq2 NB tumors (Bedoya-Reina *et al.* 2021)

	c-MYC (n=27)	c-MYC ChIP (n=34)	MYCN (n=15)
Macrophages (n=2722)	0.000	0.059	0.067
T cells (n=419)	0.000	0.000	0.000
NOR 7 (n=2985)	0.222	0.265	0.133
Undifferentiated 3 (n=6680)	0.148	0.147	0.333
NOR 9 (n=2901)	0.259	0.147	0.067
NOR 8 (n=2932)	0.444	0.235	0.067
Endothelial 4 (n=10184)	0.333	0.265	0.267
MSC 1 (n=14535)	0.111	0.235	0.133
NOR 5 (n=2091)	0.185	0.059	0.133
Undifferentiated 2 (n=19523)	0.037	0.206	0.333

Full gene set targets

Proportion of overlapping target genes: 0.0 – 0.5 – 1.0

Proportion of c-MYC/MYCN target genes with prognostic power overlapping markers in development and cancer

Figure S10

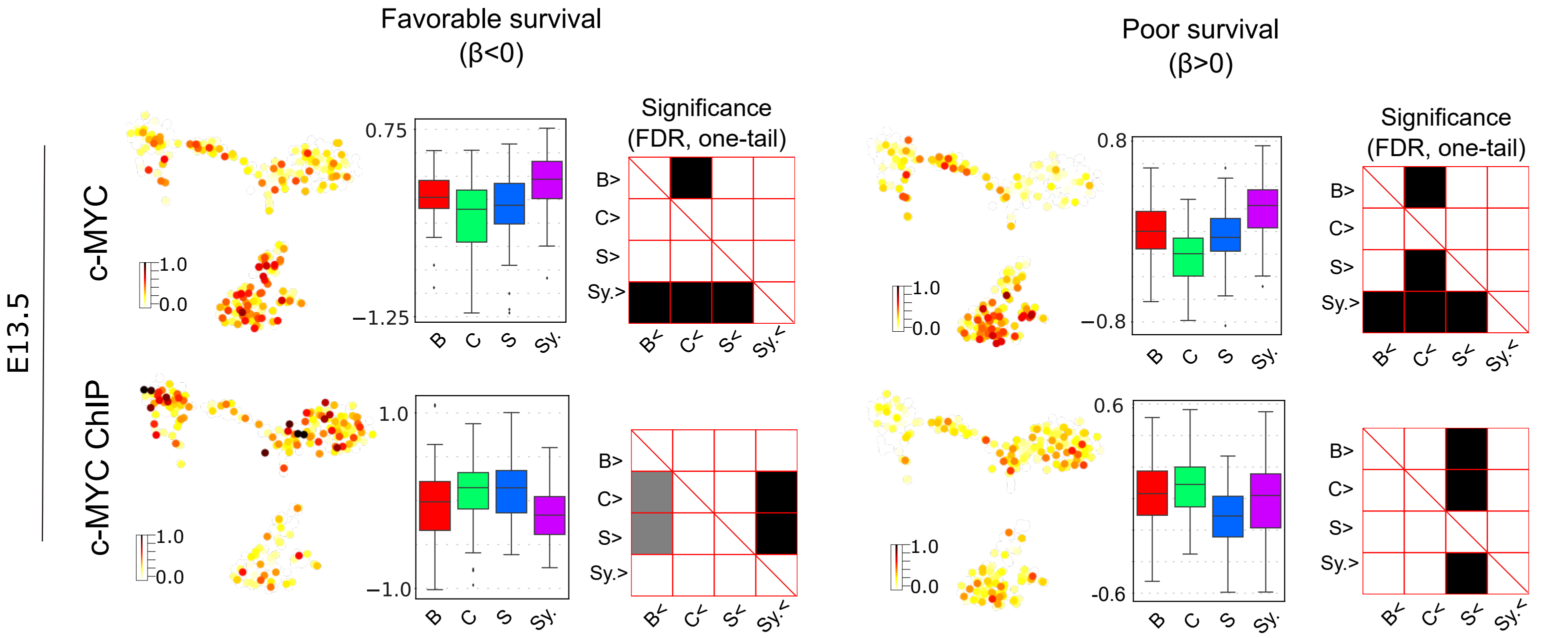

Favorable survival
(β<0)
Poor survival
(β>0)
Significance
(FDR, one-tail)
E13.5
c-MYC
c-MYC ChIP
1.0
0.0
0.75
−1.25
0.8
−0.8
1.0
−1.0
0.6
-0.6
B
C
S
Sy.
B>
C>
S>
Sy.>
B<
C<
S<
Sy.<

Figure S11

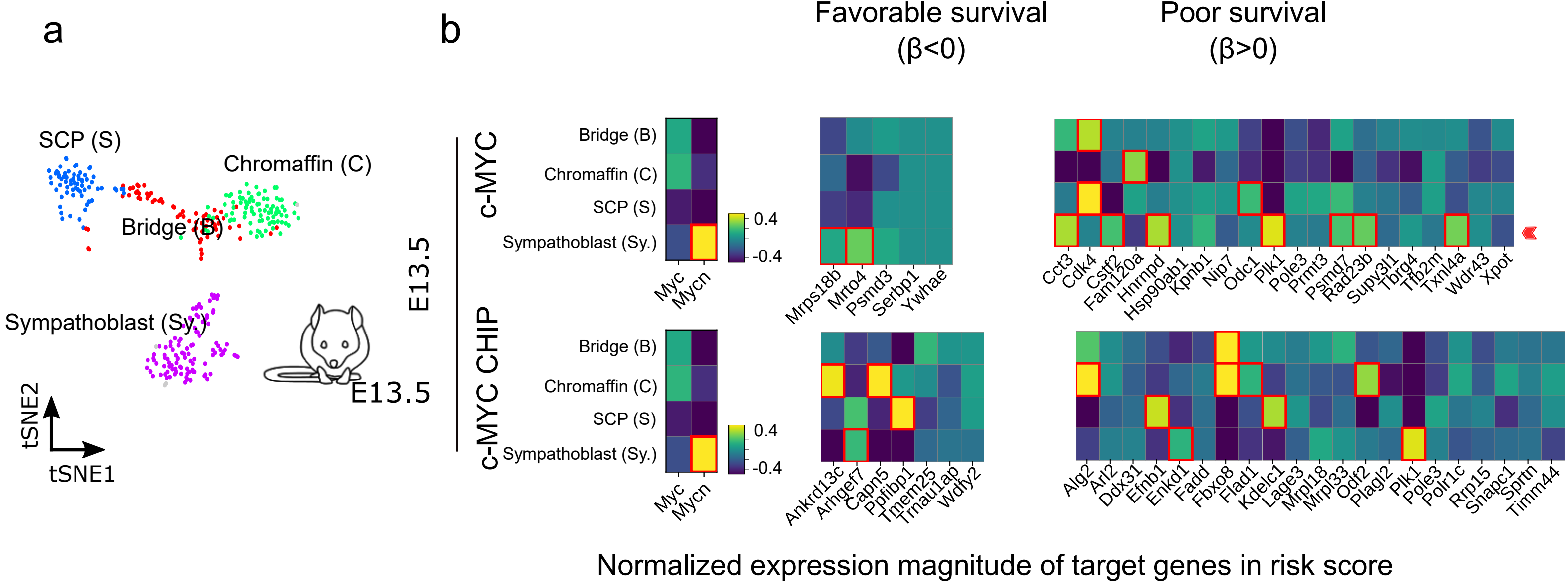
a
SCP (S)
Chromaffin (C)
Bridge (B)
Sympathoblast (Sy.)
E13.5
tSNE2
tSNE1
b
Favorable survival
(β<0)
Poor survival
(β>0)
E13.5
c-MYC
c-MYC CHIP
Bridge (B)
Chromaffin (C)
SCP (S)
Sympathoblast (Sy.)
0.4
-0.4
Myc
Mycn
Mrps18b
Mrto4
Psmd3
Serbp1
Ywhae
Cct3
Cdk4
Cstf2
Fam120a
Hnrnpd
Hsp90ab1
Kpnb1
Nip7
Odc1
Plk1
Pole3
Prmt3
Psmd7
Rad23b
Supv3l1
Tbrg4
Tfb2m
Txnl4a
Wdr43
Xpot
Ankrd13c
Arhgef7
Capn5
Ppfibp1
Tmem25
Trnau1ap
Wdfy2
Alg2
Arl2
Ddx31
Efnb1
Enkd1
Fadd
Fbxo8
Flad1
Kdelc1
Lage3
Mrpl18
Mrpl33
Odf2
Plagl2
Plk1
Pole3
Polr1c
Rrp15
Snapc1
Sprtn
Timm44
Normalized expression magnitude of target genes in risk score
