# Supplementary material for: Target Genes of c-MYC and MYCN with Prognostic Power in Neuroblastoma Exhibit Different Expressions during Sympathoadrenal Development
Source: Cancers (Basel). 2023 Sep 16;15(18):4599. doi: 10.3390/cancers15184599 (PMC10527308; doi:10.3390/cancers15184599)
Supplement: Supplementary file 1 [file cancers-15-04599-s001.zip › Supplementary_information.pdf]

Supplementary Information for

Target genes of c-MYC and MYCN with prognostic power in neuroblastoma exhibit different expressions during sympathoadrenal development

Ye Yuan [1], Mohammad Alzrigat [1], Aida Rodriguez-Garcia [1], Xueyao Wang [1], Tomas Sjöberg Bexelius [2,3], John Inge Johnsen [3], Marie Arsenian-Henriksson [1], Judit Liaño-Pons [1], and Oscar C. Bedoya-Reina [1,*]

[1] Department of Microbiology, Tumor and Cell Biology (MTC), Biomedicum, Karolinska Institutet, Stockholm, Sweden.
[2] Paediatric Oncology Unit, Astrid Lindgren's Children Hospital, Solna, Sweden,
[3] Department of Women's and Children's Health, Karolinska Institutet, Stockholm, Sweden
* Corresponding author: oscar.bedoya.reina@ki.se

Supplementary Figure S1: Validation of the prognostic performance of risk-score models in the Kocak and Versteeg cohorts. (a) Heatmaps showing the performance (AUC) of the overall survival prediction by risk scores of genes with prognostic value computed for the SEQC cohort, in the Kocak cohort [1]. The risk-score models were generated by different approaches on c-MYC/MYCN targets using the SEQC cohort [2-4]. AUC values were computed for time-dependent ROC curves. The AUCs are displayed as both values and colors of cells in the heatmap. A higher AUC value represented with bolder blue indicates a better performance of the risk score in predicting patient survival for the Kocak cohort (n=649, GSE47774) [1]. **(b)** Survival prediction power of the median risk score computed for genes with prognostic value in the Kocak cohort [1]. The risk-score models were generated by different approaches on c-MYC/MYCN targets. The color scale displays the difference in the number of observed and expected deceases in each group of patients with high- and low-median risk score, approximated by χ^2 (Chi-square). Bold red indicates a larger difference. Expected values are computed assuming that the number of deceases is the same for both groups. FDR-corrected *p*-values [5] indicate the significance of this difference, and the power of the median risk score to predict patients with different outcomes. **(c)** Validation using the Versteeg cohort (n=88, GSE 16476) [6], heatmaps display the AUC values and color-coded cells to show the effectiveness of prognostic gene-based risk scores in predicting overall survival. AUC values were computed for time-dependent ROC curves. The AUCs are displayed as both values and colors of cells in the heatmap. (d) The survival prediction power of the median risk score is displayed for the Versteeg validation cohort [6]. AUC = Area Under the Curve, ROC = Receiver Operating Characteristic, PPI= protein-protein interaction reported by the STRING database [7].

Supplementary Figure S2: Kaplan-Meier curves illustrate the predictive ability of the risk-score models for different targets in the SEQC, Kocak and Versteeg cohorts. The figure illustrates the survival differences observed between various risk groups, which were determined by dividing the cohort based on the median risk score calculated using three different target genes:

"c-MYC", "c-MYC ChIP", and "MYCN" targets.

Supplementary Figure S3: t-SNE illustration of the transcriptional similarities between patients for genes with prognostic value. t-SNE depicting the transcriptional similarities between patients for genes with prognostic value generated by different approaches on c-MYC ChIP target genes. Dots representing patients are colored following the SEQC clinical risk classification [6]: high-risk patients are in INSS stage 4 and were at least 18 months at diagnosis or with *MYCN*-amplified tumors. Patients were separated into two risk groups using the median risk-score (as detail in Material and Methods), or otherwise in the top and bottom risk score quartiles. Note that in insert **(a)**, the distribution pattern of patients is based on the clinical risk category defined by SEQC, while in the inserts **(b)** and **(c)** the distribution follows the computed risk score category generated by the LASSO-cox model.

Supplementary Figure S4: Violin-/boxplots of the risk-scores computed with the "Full gene set" of c-MYC/MYCN targets on patient groups with different clinical variables. Mann-Whitney U tests were used to assess statistical differences between groups of patients based on clinical risk factors: INSS stages (contrasting 1,2,4s with 3,4), *MYCN* amplification presence, progression status, patient outcomes, age, and gender. When comparing across the full spectrum of INSS stages (1,2,3,4, and 4s), the Kruskal-Wallis test was utilized for multi-group analysis

Supplementary Figure S5: Violin-/boxplots of the risk-scores computed for c-MYC/MYCN targets filtered with the "Without PPI" approach on patient groups with different clinical variables. Significance was computed using Mann-Whitney U or Kruskall-Wallis test (for multiple groups).

Supplementary Figure S6: Violin-/boxplots of the risk-scores computed for c-MYC/MYCN targets filtered with the "With PPI" approach on groups of patients with different clinical variables. Significance was computed using Mann-Whitney U or Kruskall-Wallis test (for multiple groups).

Supplementary Figure S7: c-MYC/MYCN directly binds to the Transcription Start Site (TSS) of c-MYC, c-MYC ChIP, and MYCN target genes. Genome-browser representation of c-MYC ChIP-Seq at the TSS of the *ODC1* **(a)** and *RAD50* **(b)** in the MYCN-non-amplified NB69 and SKNAS cell lines. **(c)** Genome-browser representation of MYCN ChIP-Seq at TSS of the *DKC1* (MYCN target gene) in the *MYCN*-amplified KELLY and NGP cell lines. **(d)** Multiple occurrence of E-boxes in the promoter region of *ODC1* (c-MYC target gene), *RAD50* (c-MYC ChIP-Seq target gene), *DKC1* (MYCN target gene) are highlighted. *ODC1*= Ornithine Decarboxylase 1; *RAD50*= *RAD50* Double Strand Break Repair Protein; *DKC1* = Dyskerin pseudouridine synthase 1; TSS=transcription start site. The Figure was adapted from the R2 genome browser (http://r2.amc.nl), using Maris et al., 2019 data set [8] on the human genome assembly hg19.

Supplementary Figure S8: Overlapping proportion of c-MYC/MYCN target genes (obtained from the SEQC dataset) and markers for different cell clusters during sympathoadrenal development and in NB. Proportion (i.e., frequency) of overlapping genes with prognostic power

obtained by 1) c-MYC/MYCN gene targets ("Full gene set", x-axis), and 2) markers of different cells clusters during **(a)** mouse and **(b)** human sympathoadrenal development, and **(c-e)** in NB tumor. The number of genes with significant prognostic value in each cluster is displayed in parentheses. The cells display in colors the pairs of gene sets for which the number of patients is significantly higher than expected by chance (Fisher's exact test, one-tail, FDR<0.05 in red, and FDR<0.01 in yellow). * = Significance obtained with less than three overlapping genes.

Supplementary Figure S9: Overlapping proportion of c-MYC/MYCN target genes (obtained from the SEQC dataset) and markers with predictive power for different cell clusters during sympathoadrenal development and in NB. Proportion (i.e., frequency) of overlapping genes with prognostic power by 1) c-MYC/MYCN targets ("Full gene set", x-axis), and 2) markers of different cells clusters during **(a)** mouse and **(b)** human sympathoadrenal development, and **(c-e)** in NB tumor. The number of genes with significant prognostic value in each cluster is displayed in parentheses. The cells display in colors the pairs of gene sets for which the number of genes is significantly higher than expected by chance (Fisher's exact test, one-tail, FDR<0.05 in red, and FDR<0.01 in yellow).

Supplementary Figure S10: Signature scores of c-MYC/MYCN targets with prognostic value during mouse sympathoadrenal development at E13.5. Signature scores of c-MYC/MYCN targets with worse ($\beta<0$) and better ($\beta>0$) prognostic value in cells of the murine developing sympathodarenal anlagen at E13.5: B (bridge), C (chromaffin), S (SCPs), and Sy. (Sympathoblast) [9]. A greater signature score indicates a larger average expression of c-MYC/MYCN targets than expected by chance. Boxplots illustrate the distribution of signature scores for targets in single-cell clusters. Significance in pairwise comparisons is displayed in the adjunct matrix plots. Filled cells indicate that clusters in the y-axis present a significantly higher signature score than those in the x-axis (Mann-Whitney U test, one-tail, FDR). FDR values < 0.05 are displayed in gray and < 0.01 in black.

Supplementary Figure S11: Expression of c-MYC/MYCN targets with favorable ($\beta<0$) and poor prognostic ($\beta>0$) value during mouse sympathoadrenal development at E13.5. (a, b) Average expressions of *MYC* (encoding c-MYC) and c-MYC targets with prognostic value were calculated for single cell clusters during sympathoadrenal development. The heatmaps illustrate the average normalized expression magnitude as computed by PAGODA [10] for c-MYC targets with favorable ($\beta<0$) and poor prognostic ($\beta>0$) value during mouse sympathoadrenal development. Cells surrounded by a red square indicates genes significantly upregulated in cell clusters during development (Welch t-test, one-tail, FDR<0.01). A red arrow signals the enrichment of target genes with favorable ($\beta<0$) or poor ($\beta>0$) prognostic value in cell clusters during development. The expression magnitude displayed is truncated to ranges between -0.4 and 0.4.

Supplementary Table S1: Unfiltered c-MYC/MYCN target sets included for the analysis. The gene lists comprise c-MYC [11], c-MYC ChIP [12], and MYCN target sets [13].

Supplementary Table S2: Target genes before LASSO-cox screening. The gene lists were generated after differential expression screening or uniCox regression screening, as illustrated in

Figure 1 and detailed in Methods.

Supplementary Table S3: **Target genes from different approaches.** The gene lists were generated after different filtered approaches illustrated in Figure 1 and detailed in Methods. Genes without expression reported for the Kocak validation cohort are indicated with a star (*)[1], and for Versteeg validation cohort are indicated with dollar sign (**$**) [6]. Genes lacking high-confidence 1:1 orthologue between human and mouse are indicated with a plus (+). Genes absent in E12.5/E13.5 murine developing data set are indicated by a section sign (§). Genes with prognostic value also found after correcting for response to therapy, as well as for other clinical variables (as detailed in Materials and Methods).

References

1. Kocak, H.; Ackermann, S.; Hero, B.; Kahlert, Y.; Oberthuer, A.; Juraeva, D.; Roels, F.; Theissen, J.; Westermann, F.; Deubzer, H.; et al. Hox-C9 activates the intrinsic pathway of apoptosis and is associated with spontaneous regression in neuroblastoma. *Cell Death Dis* **2013**, *4*, e586, doi:10.1038/cddis.2013.84.
2. Consortium, S.M.-I. A comprehensive assessment of RNA-seq accuracy, reproducibility and information content by the Sequencing Quality Control Consortium. *Nat Biotechnol* **2014**, *32*, 903-914, doi:10.1038/nbt.2957.
3. Su, Z.; Fang, H.; Hong, H.; Shi, L.; Zhang, W.; Zhang, W.; Zhang, Y.; Dong, Z.; Lancashire, L.J.; Bessarabova, M.; et al. An investigation of biomarkers derived from legacy microarray data for their utility in the RNA-seq era. *Genome Biol* **2014**, *15*, 523, doi:10.1186/s13059-014-0523-y.
4. Zhang, W.; Yu, Y.; Hertwig, F.; Thierry-Mieg, J.; Zhang, W.; Thierry-Mieg, D.; Wang, J.; Furlanello, C.; Devanarayan, V.; Cheng, J.; et al. Comparison of RNA-seq and microarray-based models for clinical endpoint prediction. *Genome Biol* **2015**, *16*, 133, doi:10.1186/s13059-015-0694-1.
5. Benjamini, Y.; Hochberg, Y. Controlling the false discovery rate: A practical and powerful approach to multiple testing. *Journal of the Royal Statistical Society: Series B (Methodological)* **2018**, *57*, 289-300, doi:10.1111/j.2517-6161.1995.tb02031.x.
6. Molenaar, J.J.; Koster, J.; Zwijnenburg, D.A.; van Sluis, P.; Valentijn, L.J.; van der Ploeg, I.; Hamdi, M.; van Nes, J.; Westerman, B.A.; van Arkel, J.; et al. Sequencing of neuroblastoma identifies chromothripsis and defects in neuritogenesis genes. *Nature* **2012**, *483*, 589-593, doi:10.1038/nature10910.
7. Szklarczyk, D.; Kirsch, R.; Koutrouli, M.; Nastou, K.; Mehryary, F.; Hachilif, R.; Gable, A.L.; Fang, T.; Doncheva, N.T.; Pyysalo, S.; et al. The STRING database in 2023: protein-protein association networks and functional enrichment analyses for any sequenced genome of interest. *Nucleic Acids Res* **2023**, *51*, D638-D646, doi:10.1093/nar/gkac1000.
8. Upton, K.; Modi, A.; Patel, K.; Kendsersky, N.M.; Conkrite, K.L.; Sussman, R.T.; Way, G.P.; Adams, R.N.; Sacks, G.I.; Fortina, P.; et al. Epigenomic profiling of neuroblastoma cell lines. *Sci Data* **2020**, *7*, 116, doi:10.1038/s41597-020-0458-y.
9. Furlan, A.; Dyachuk, V.; Kastriti, M.E.; Calvo-Enrique, L.; Abdo, H.; Hadjab, S.; Chontorotzea, T.; Akkuratova, N.; Usoskin, D.; Kamenev, D.; et al. Multipotent peripheral glial cells generate neuroendocrine cells of the adrenal medulla. *Science* **2017**, *357*, doi:10.1126/science.aal3753.

10. Fan, J.; Salathia, N.; Liu, R.; Kaeser, G.E.; Yung, Y.C.; Herman, J.L.; Kaper, F.; Fan, J.B.; Zhang, K.; Chun, J.; et al. Characterizing transcriptional heterogeneity through pathway and gene set overdispersion analysis. *Nat Methods* **2016**, *13*, 241-244, doi:10.1038/nmeth.3734.
11. Liberzon, A.; Birger, C.; Thorvaldsdottir, H.; Ghandi, M.; Mesirov, J.P.; Tamayo, P. The Molecular Signatures Database (MSigDB) hallmark gene set collection. *Cell Syst* **2015**, *1*, 417-425, doi:10.1016/j.cels.2015.12.004.
12. Kim, J.; Lee, J.H.; Iyer, V.R. Global identification of Myc target genes reveals its direct role in mitochondrial biogenesis and its E-box usage in vivo. *PLoS One* **2008**, *3*, e1798, doi:10.1371/journal.pone.0001798.
13. Valentijn, L.J.; Koster, J.; Haneveld, F.; Aissa, R.A.; van Sluis, P.; Broekmans, M.E.; Molenaar, J.J.; van Nes, J.; Versteeg, R. Functional MYCN signature predicts outcome of neuroblastoma irrespective of MYCN amplification. *Proc Natl Acad Sci U S A* **2012**, *109*, 19190-19195, doi:10.1073/pnas.1208215109.
